# Crystal structures of four isomeric hydrogen-bonded co-crystals of 6-methyl­quinoline with 2-chloro-4-nitro­benzoic acid, 2-chloro-5-nitro­benzoic acid, 3-chloro-2-nitro­benzoic acid and 4-chloro-2-nitro­benzoic acid

**DOI:** 10.1107/S2056989020013134

**Published:** 2020-10-06

**Authors:** Kazuma Gotoh, Hiroyuki Ishida

**Affiliations:** a Department of Chemistry, Faculty of Science, Okayama University, Okayama 700-8530, Japan

**Keywords:** crystal structure, 2-chloro-4-nitro­benzoic acid, 2-chloro-5-nitro­benzoic acid, 3-chloro-2-nitro­benzoic acid, 4-chloro-2-nitro­benzoic acid, 6-methyl­quinoline, hydrogen bond, disorder, Hirshfeld surface

## Abstract

The structures of the four isomeric compounds of 6-methyl­quinoline with chloro- and nitro-substituted benzoic acids, C_7_H_4_ClNO_4_·C_10_H_9_N, namely, 2-chloro-4-nitro­benzoic acid–6-methyl­quinoline (1/1), (I), 2-chloro-5-nitro­benzoic acid–6-methyl­quinoline (1/1), (II), 3-chloro-2-nitro­benzoic acid–6-methyl­quinoline (1/1), (III), and 4-chloro-2-nitro­benzoic acid–6-methyl­quinoline (1/1), (IV), have been determined at 185–190 K. In each compound, the acid and base mol­ecules are linked by a short hydrogen bond between a carboxyl O atom and an N atom of the base. The O⋯N distances are 2.5452 (12), 2.6569 (13), 2.5640 (17) and 2.514 (2) Å, respectively, for compounds (I)–(IV). In the hydrogen-bonded acid–base units of (I), (III) and (IV), the H atoms are each disordered over two positions with O site:N site occupancies of 0.65 (3):0.35 (3), 0.59 (4):0.41 (4) and 0.48 (5):0.52 (5), respectively, for (I), (III) and (IV). The H atom in the hydrogen-bonded unit of (II) is located at the O-atom site. In all of the crystals of (I)–(IV), π–π inter­actions between the quinoline ring system and the benzene ring of the acid mol­ecule are observed. In addition, a π–π inter­action between the benzene rings of adjacent acid mol­ecules and a C—H⋯O hydrogen bond are observed in the crystal of (I), and C—H⋯O hydrogen bonds and O⋯Cl contacts occur in the crystals of (III) and (IV). These inter­molecular inter­actions connect the acid and base mol­ecules, forming a layer structure parallel to the *bc* plane in (I), a column along the *a*-axis direction in (II), a layer parallel to the *ab* plane in (III) and a three-dimensional network in (IV). Hirshfeld surfaces for the title compounds mapped over *d*_norm_ and shape index were generated to visualize the weak inter­molecular inter­actions.

## Abstract 2

The structures of the four isomeric hydrogen-bonded 1:1 co-crystals of 6-methyl­quinoline with 2-chloro-4-nitro­benzoic acid, 2-chloro-5-nitro­benzoic acid, 3-chloro-2-nitro­benzoic acid and 4-chloro-2-nitro­benzoic acid have been determined at 185–190 K. In each crystal, the acid and base mol­ecules are linked by a short O—H⋯N hydrogen bond.

## Chemical context   

Properties of hydrogen bonds formed between organic acids and organic bases depend on the p*K*_a_ values of the acids and bases as well as inter­molecular inter­actions in the crystals. In our ongoing study on crystal structures of the system of quinoline derivatives–chloro- and nitro-substituted benzoic acids, we have shown that three compounds of quinoline with 3-chloro-2-nitro­benzoic acid, 4-chloro-2-nitro­benzoic acid and 5-chloro-2-nitro­benzoic acid, the Δp*K*_a_ [p*K*_a_(base) − p*K*_a_(acid)] values of which are 3.08, 2.93 and 3.04, respectively, have a short double-well O—H⋯N/O⋯H—N hydrogen bond between the carb­oxy O atom and the aromatic N atom (Gotoh & Ishida, 2009 ▸). On the other hand, in 2-chloro-5-nitro­benzoic acid–quinoline (1/1) (Δp*K*_a_ = 2.68; Gotoh & Ishida, 2009 ▸), 2-chloro-4-nitro­benzoic acid–quinoline (1/1) (Δp*K*_a_ = 2.86; Gotoh & Ishida, 2011 ▸), 3-chloro-2-nitro­benzoic acid–6-nitro­quinolune (1/1) (Δp*K*_a_ = 1.42), 8-hy­droxy­quinolinium 3-chloro-2-nitro­benzoate (Δp*K*_a_ = 3.02) and 3-chloro-2-nitro­benzoic acid–5-nitro­quinoline (1/1) (Δp*K*_a_ = 0.98) (Gotoh & Ishida, 2019*a* ▸), 2-chloro-4-nitro­benzoic acid–5-nitro­quinoline (1/1) (Δp*K*_a_ = 0.76), 5-chloro-2-nitro­benzoic acid–5-nitro­quinoline (1/1) (Δp*K*_a_ = 0.94) (Gotoh & Ishida, 2019*b* ▸), such a short disordered hydrogen bond was not observed. We report here crystal structures of title four isomeric compounds, namely, 2-chloro-4-nitro­benzoic acid–6-methyl­quinoline (1/1), (I), 2-chloro-5-nitro­benzoic acid–6-methyl­quinoline (1/1), (II), 3-chloro-2-nitro­benzoic acid–6-methyl­quinoline (1/1), (III), and 4-chloro-2-nitro­benzoic acid–6-methyl­quinoline (1/1), (IV), in order to extend our studies of short hydrogen bonding and weak inter­molecular inter­actions in the system of quinoline derivatives–chloro- and nitro-substituted benzoic acids. The Δp*K*_a_ values are 3.16, 2.98, 3.38 and 3.23, respectively, for (I)–(IV).
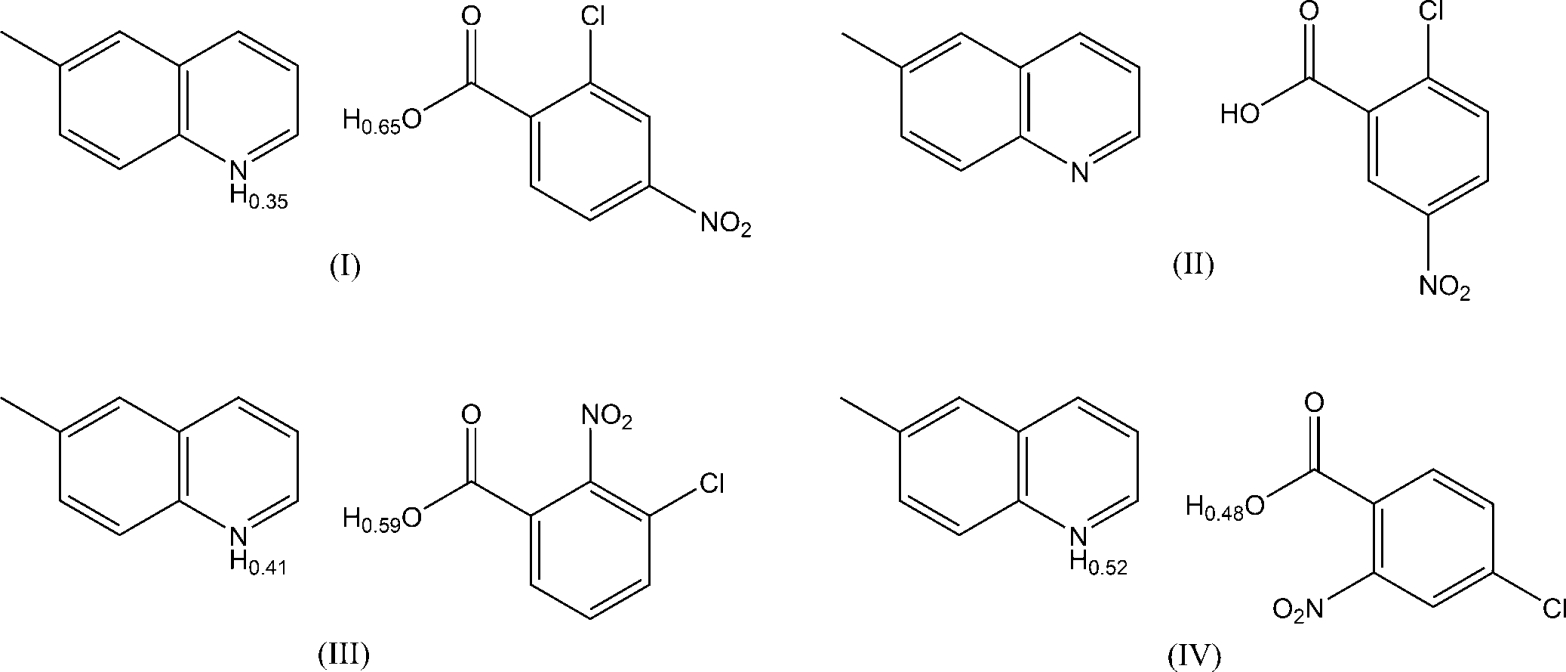


## Structural commentary   

**Figure 1:**
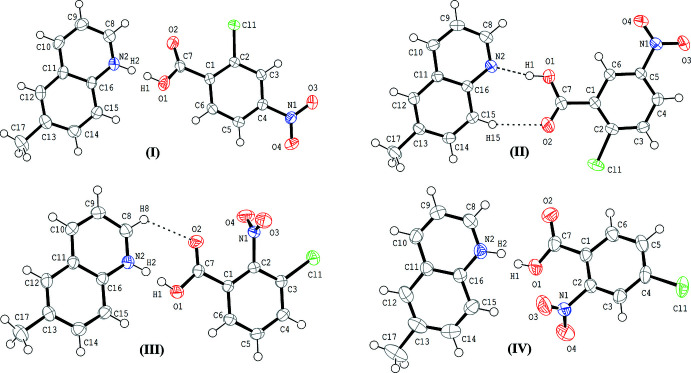
Mol­ecular structures of the title compounds (I)–(IV), showing the atom-numbering scheme. Displacement ellipsoids are drawn at the 50% probability level and H atoms are shown as small spheres of arbitrary radii. In the hydrogen bonds between the carb­oxy group and the N atom of the base of compounds (I), (III) and (IV), the H atoms are each disordered over two positions. Dashed lines in (II) and (III) indicate the O—H⋯N and C—H⋯O hydrogen bonds.

The mol­ecular structures of compounds (I)–(IV) are shown in Fig. 1 ▸. In each compound, the acid and base mol­ecules are linked by a hydrogen bond between the carb­oxy group and the N atom of the base. In (I), (III) and (IV), short hydrogen bonds are observed with N⋯O distances of 2.5452 (12), 2.5640 (17) and 2.515 (2) Å, respectively. (Tables 1 ▸, 3 ▸ and 4 ▸). In these hydrogen bonds, the H atoms are each disordered over two sites; the occupancies of the O site and the N site refined to 0.65 (3) and 0.35 (3), 0.59 (4) and 0.41 (4), and 0.48 (5) and 0.52 (5), respectively, for (I), (III) and (IV). In (II), the H atom in the hydrogen bond is located at the O site with an N⋯O distance of 2.6569 (13) Å (Table 2 ▸), being longer than those in (I), (III) and (IV). Weak C—H⋯O hydrogen bonds are each observed in the acid–base unit of (II) (C15—H15⋯O2; Table 2 ▸) and the unit of (III) (C8—H8⋯O2; Table 3 ▸).

In the hydrogen-bonded acid–base unit of compound (I), the quinoline ring system (N2/C8–C16) and the benzene ring (C1–C6) are almost coplanar with a dihedral angle of 1.11 (4)°, while the quinoline ring system and the carb­oxy group (O1/C7/O2) of the acid are twisted to each other with a dihedral angle of 28.59 (12)°. In the acid mol­ecule, the benzene ring makes dihedral angles of 29.36 (12) and 8.24 (11)°, respectively, with the carb­oxy group and the nitro group (O3/N1/O4).

Similar to (I), the quinoline ring system (N2/C8–C16) in the hydrogen-bonded acid–base unit of (II) makes dihedral angles of 2.15 (4) and 24.51 (15)°, respectively, with the benzene ring and the carb­oxy group. The benzene ring makes dihedral angles of 22.63 (15) and 0.77 (14)°, respectively, with the carb­oxy group and the nitro group.

Compound (III) crystallizes in the non-centrosymmetric space group *P*2_1_2_1_2_1_. In the acid–base unit, the quinoline ring system and the benzene ring of the acid are slightly twisted to each other with a dihedral angle of 14.50 (5)°. The quinoline ring system and the carb­oxy group are also slightly twisted with a dihedral angle of 12.55 (18)°. The benzene ring makes dihedral angles of 3.14 (18) and 85.04 (11)°, respectively, with the carb­oxy group and the nitro group.

Compound (IV) crystallizes in the non-centrosymmetric space group *Cc*. In the acid–base unit, the quinoline ring system and the benzene ring of the acid are twisted to each other with a dihedral angle of 30.39 (9)°. The quinoline ring system and the carb­oxy group are also twisted with a dihedral angle of 21.7 (3)°. The benzene ring makes dihedral angles of 16.4 (3) and 74.4 (3)°, respectively, with the carb­oxy group and the nitro group.

## Supra­molecular features   

**Figure 2:**
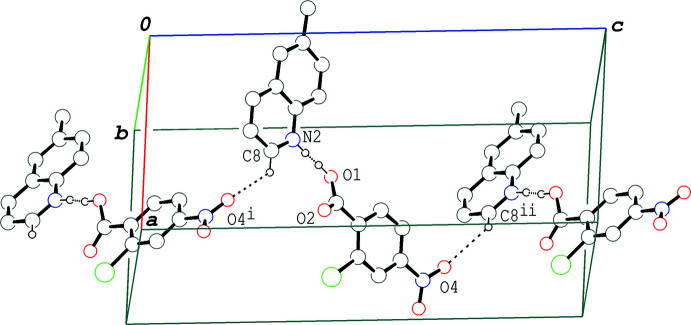
A packing diagram of (I), showing the hydrogen-bonded chain structure formed *via* the O—H⋯N/O⋯·H—N and C—H⋯O hydrogen bonds (dashed lines). H atoms not involved in the hydrogen bonds are omitted for clarity. Symmetry codes: (i) *x*, −*y* + 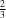, *z* − 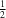; (ii) *x*, −*y* + 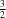, −*z* + 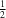.

**Figure 3:**
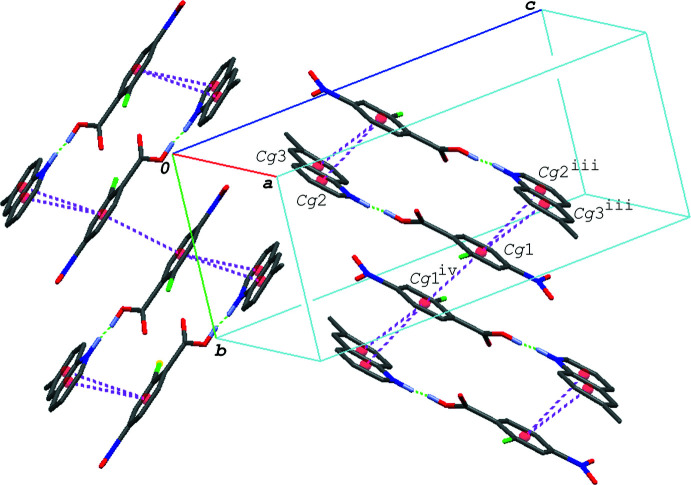
A packing diagram of (I), showing the column structure formed *via* the π–π inter­actions (magenta dashed lines). H atoms except for in the O—H⋯N/O⋯·H—N hydrogen bonds (green dashed lines) are omitted for clarity. *Cg*1, *Cg*2 and *Cg*3 are the centroids of the C1–C6, N2/C8–C11/C16 and C11–C16 rings, respectively. Symmetry codes: (iii) −*x* + 1, −*y* + 1, −*z* + 1; (iv) −*x* + 1, −*y* + 2, −*z* + 1.

In the crystal of (I), the hydrogen–bonded acid-base units are linked by a C—H⋯O hydrogen bond (C8—H8⋯O4^i^; symmetry code as given in Table 1 ▸), forming a zigzag chain propagating along the *c-*axis direction (Fig. 2 ▸). The acid–base units, which are related to each other by an inversion center, are linked together *via* π–π inter­actions between the quinoline ring system and the benzene ring of the acid mol­ecule, forming a centrosymmetric dimeric unit (Fig. 3 ▸); the centroid–centroid distances are 3.7217 (6) and 3.7216 (6) Å, respectively, for *Cg*1⋯*Cg*2^iii^ and *Cg*1⋯*Cg*3^iii^, where *Cg*1, *Cg*2 and *Cg*3 are the centroids of the C1–C6, N2/C8–C11/C16 and C11–C16 rings, respectively [symmetry code: (iii) −*x* + 1, −*y* + 1, −*z* + 1]. The dimeric units are further linked into a column structure stacked along the *b-*axis direction through a weak π–π inter­action between the benzene rings with *Cg*1⋯*Cg*1^iv^ = 3.9401 (6) Å [symmetry code: (iv) −*x* + 1, −*y* + 2, −*z* + 1]. The mol­ecular chains are thus stacked into a layer parallel to the *bc* plane *via* these π–π inter­actions.

**Figure 4:**
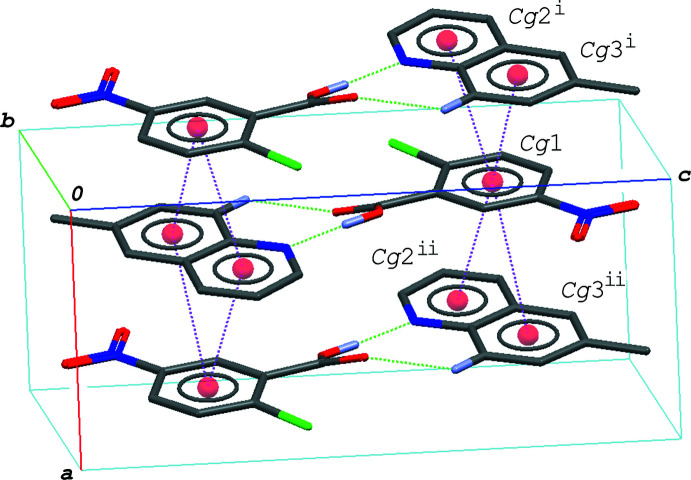
A packing diagram of (II), showing the column structure formed *via* the π–π inter­actions (magenta dashed lines). H atoms not involved in the O—H⋯N and C—H⋯O hydrogen bonds (green dashed lines) are omitted for clarity. *Cg*1, *Cg*2 and *Cg*3 are the centroids of the C1–C6, N2/C8–C11/C16 and C11–C16 rings, respectively. Symmetry codes: (i) −*x*, −*y* + 1, −*z* + 1; (ii) −*x* + 1, −*y* + 1, −*z* + 1.

In the crystal of (II), the acid and base mol­ecules are alternately stacked in a column *via* π–π inter­actions between the acid benzene ring and the quinoline ring system, so that the hydrogen-bonded acid–base units related by an inversion center are linked into a column structure along the *a-*axis direction (Fig. 4 ▸). The centroid–centroid distances are 3.6438 (6), 3.5745 (6), 3.6560 (6) and 3.7375 (6) Å, respectively, for *Cg*1⋯*Cg*2^i^, *Cg*1⋯*Cg*2^ii^, *Cg*1⋯*Cg*3^i^ and *Cg*1⋯*Cg*3^ii^, where *Cg*1, *Cg*2 and *Cg*3 are the centroids of the C1–C6, N2/C8–C11/C16 and C11–C16 rings, respectively [symmetry codes: (i) −*x*, −*y* + 1, −*z* + 1; (ii) −*x* + 1, −*y* + 1, −*z* + 1]. There are no significant inter­actions between the columns.

**Figure 5:**
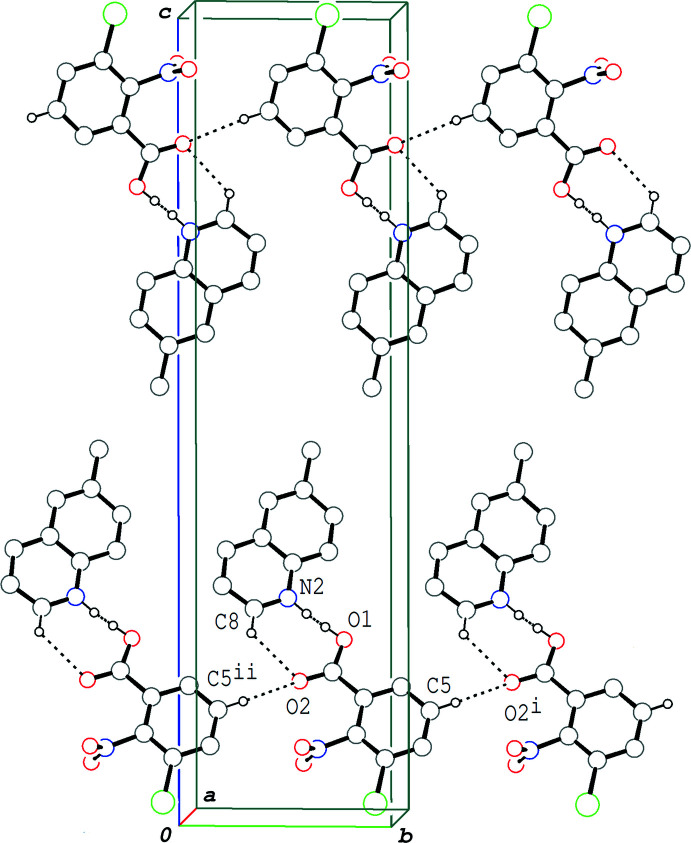
A packing diagram of (III), showing two tape structures (top and bottom) related by an inversion symmetry to each other, formed by O—H⋯N/O⋯·H—N and C—H⋯O hydrogen bonds (dashed lines). H atoms not involved in the hydrogen bonds are omitted for clarity. Symmetry codes: (i) *x*, *y* + 1, *z*; (ii) *x*, *y* − 1, *z*.

**Figure 6:**
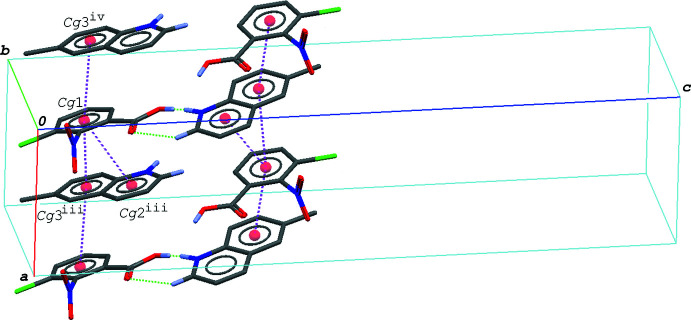
A packing diagram of (III), showing the column structure formed *via* the π–π inter­actions (magenta dashed lines). H atoms not involved in the O—H⋯·N/O⋯H—N and C—H⋯O hydrogen bonds (green dashed lines) are omitted for clarity. *Cg*1, *Cg*2 and *Cg*3 are the centroids of the C1–C6, N2/C8–C11/C16 and C11–C16 rings, respectively. Symmetry codes: (iii) −*x* + 1, *y* + 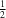, −*z* + 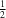; (iv) −*x*, *y* + 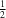, −*z* + 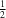.

In the crystal of (III), the hydrogen-bonded acid–base units are linked by a C—H⋯O hydrogen bond (C5—H5⋯O2^i^; symmetry code as in Table 3 ▸), forming a tape structure propagating along the *b-*axis direction (Fig. 5 ▸). The acid and base mol­ecules are alternately stacked in a column along the *a* axis direction *via* π–π inter­actions between the acid ring and the quinoline ring system (Fig. 6 ▸), and thus the hydrogen-bonded acid–base units form a layer lying parallel to the *ab* plane. The centroid–centroid distances are 3.6415 (8), 3.6126 (8) and 3.6393 (8) Å, respectively, for *Cg*1⋯*Cg*2^iii^, *Cg*1⋯*Cg*3^iii^ and *Cg*1⋯*Cg*3^iv^, where *Cg*1, *Cg*2 and *Cg*3 are the centroids of the C1–C6, N2/C8–C11/C16 and C11–C16 rings, respectively [symmetry codes: (iii) −*x* + 1, *y* + 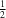, −*z* + 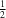; (iv) −*x*, *y* + 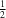, −*z* + 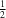]. A short O⋯Cl contact [O3⋯Cl1^v^ = 3.0934 (14) Å; symmetry code: (v) *x* − 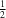, −*y* + 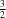, −*z*] is observed between the layers.

**Figure 7:**
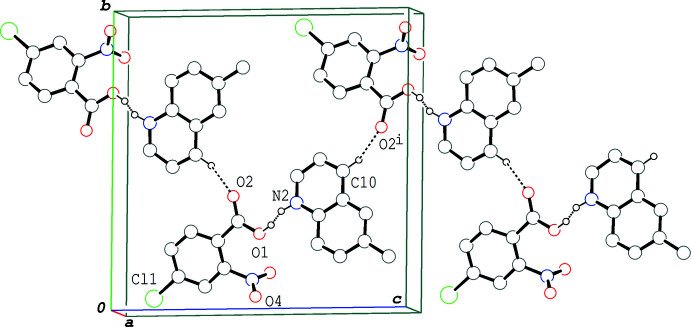
A packing diagram of (IV), showing the zigzag chain structure along the *c* axis *via* O—H⋯N/O⋯·H—N and C—H⋯O hydrogen bonds. H atoms not involved in the hydrogen bonds are omitted for clarity. Symmetry code: (i) *x*, −*y* + 1, *z* + 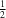.

**Figure 8:**
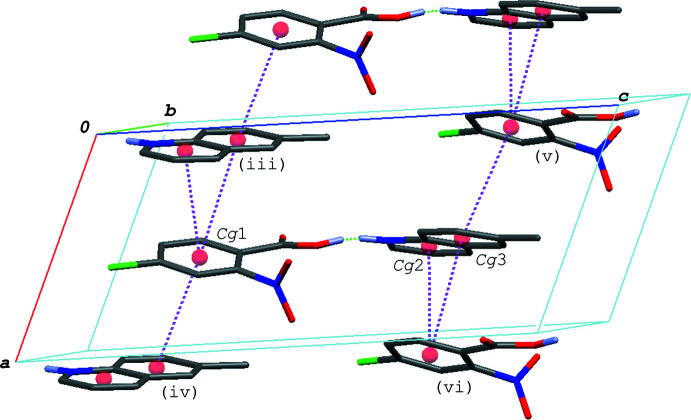
A packing diagram of (IV), showing the column structure formed *via* the π–π inter­actions (magenta dashed lines). H atoms not involved in the O—H⋯·N/O⋯H—N hydrogen bonds (green dashed lines) are omitted for clarity. *Cg*1, *Cg*2 and *Cg*3 are the centroids of the C1–C6, N2/C8–C11/C16 and C11–C16 rings, respectively. Symmetry codes: (iii) *x* − 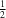, −*y* + 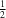, *z* − 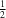; (iv) *x* + 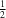, −*y* + 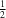, *z* − 1/2: (v) *x* − 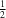, −*y* + 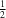, *z* + 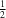; (vi) *x* + 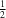, −*y* + 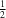, *z* + 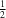.

In the crystal of (IV), the hydrogen-bonded acid–base units are linked into a zigzag chain structure propagating along the *c-*axis direction (Fig. 7 ▸) *via* C—H⋯O hydrogen bonds (C10—H10⋯O2^i^; symmetry code as in Table 4 ▸). The chains are further linked into a sheet parallel to the *bc* plane *via* an O⋯Cl short contact [O4⋯Cl1^ii^ = 3.017 (3) Å; (ii) *x*, −*y*, *z* + 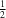]. Similar to (III), the acid and base mol­ecules are alternately stacked in a column along the *a-*axis direction *via* π–π inter­actions between the acid ring and the quinoline ring system (Fig. 8 ▸), and thus the above sheets form a three-dimensional network. The centroid–centroid distances are 3.5813 (13), 3.7987 (14) and 3.7382 (14) Å, respectively, for *Cg*1⋯*Cg*2^iii^, *Cg*1⋯*Cg*3^iii^ and *Cg*1⋯*Cg*3^iv^, where *Cg*1, *Cg*2 and *Cg*3 are the centroids of the C1–C6, N2/C8–C11/C16 and C11–C16 rings, respectively [symmetry codes: (iii) *x* − 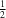, −*y* + 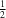, *z* − 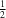; (iv) *x* + 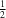, −*y* + 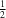, *z* − 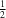].

**Figure 9:**
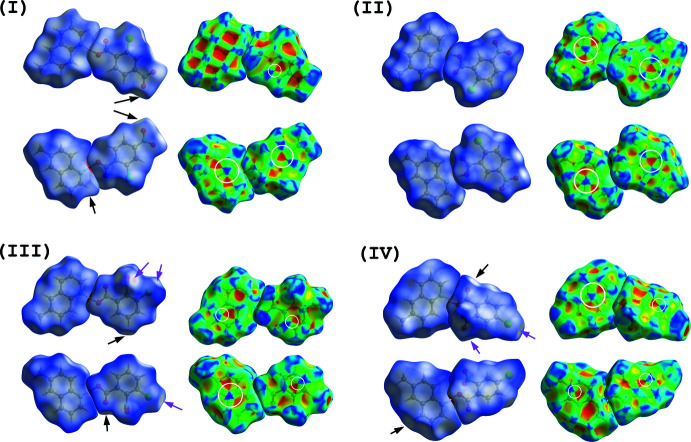
Hirshfeld surfaces [front (top) and back (bottom) views] for the compounds of (I)–(IV) mapped over *d*_norm_ and shape index, indicating the C—H⋯O inter­actions (black arrows), O⋯Cl contacts (magenta arrows) and π–π inter­actions (white circles).

Hirshfeld surfaces for compounds (I)–(IV) mapped over *d*_norm_ and shape index (Turner *et al.*, 2017 ▸; McKinnon *et al.*, 2004 ▸, 2007 ▸) are shown in Fig. 9 ▸. The C—H⋯O inter­actions in (I), (III) and (IV) are viewed as faint-red spots on the *d*_norm_ surfaces (black arrows in Fig. 9 ▸). In addition to these inter­actions, the O⋯Cl contacts in (III) and (IV) are shown as faint-red spots (magenta arrows). The π–π inter­actions between the acid ring and the quinoline ring system in (I)–(IV) are indicated by blue and red triangles on the shape index surfaces (white circles in Fig. 9 ▸).

## Database survey   

A search of the Cambridge Structural Database (Version 5.41, last update May 2020; Groom *et al.*, 2016 ▸) for organic co-crystals/salts of 6-methyl­quinoline with carb­oxy­lic acid derivatives showed two structures, namely, 6-methyl­quinoline hemikis(*trans*-but-2-enedioic acid) (Cambridge Structural Database refcode LASGUJ; Bekö *et al.*, 2012 ▸), sesquikis(6-methyl­quinoline) hemikis(quinoline) *trans*-but-2-enedioic acid (LASHAQ; Beko *et al.*, 2012 ▸). A search for organic co-crystals/salts of 2-chloro-4-nitro­benzoic acid, 2-chloro-5-nitro­benzoic acid, 3-chloro-2-nitro­benzoic acid and 4-chloro-2-nitro­benzoic acid gave 61, 12, 9 and 9 structures, respectively. Limiting the search for quinoline derivatives of these compounds gave 3, 2, 4 and 2 compounds, namely, for 2-chloro-4-nitro­benzoic acid: 2-chloro-4-nitro­benzoic acid–5-nitro­quinoline (NUBHEA; Gotoh & Ishida, 2019*b* ▸), 8-hy­droxy­quinolinium 2-chloro-4-nitro­benzoate (WOPDEM; Babu & Chandrasekaran, 2014 ▸), 2-chloro-4-nitro­benzoic acid–quinoline (1/1) (YAGFAP; Gotoh & Ishida, 2011 ▸), for 2-chloro-5-nitro­benzoic acid: 2-chloro-5-nitro­benzoic acid–quinoline (1/1) (AJIWIA; Gotoh & Ishida, 2009 ▸), 8-hy­droxy-2-methyl­quinolinium 2-chloro-5-nitro­benzoate dihydrate (HIHPIY; Tan, 2007 ▸), for 3-chloro-2-nitro­benzoic acid: 3-chloro-2-nitro­benzoic acid–quinoline (1/1) (AJIWOG, Gotoh & Ishida, 2009 ▸), 3-chloro-2-nitro­benzoic acid–5-nitro­quinoline (1/1) (XOWVUD; Gotoh & Ishida, 2019*a* ▸), 3-chloro-2-nitro­benzoic acid–6-nitro­quinoline (1/1) (XOWWAK, Gotoh & Ishida, 2019*a* ▸), 8-hy­droxy­quinolin-1-ium 3-chloro-2-nitro­benzoate (XOWWEO; Gotoh & Ishida, 2019*a* ▸), and for 4-chloro-2-nitro­benzoic acid: 4-chloro-2-nitro­benzoic acid–quinoline (AJIWUM; Gotoh & Ishida, 2009 ▸), 4-hy­droxy­quinolin-1-ium 4-chloro-2-nitro­benzoate (WOVZOZ; Gotoh & Ishida, 2019*c* ▸). Of these compounds, AJIWOG and AJIWUM show disordered O—H⋯N/O⋯H—N hydrogen bonds, while WOVZOZ shows a disorder structure in the O—H⋯O hydrogen bond accompanied by a keto–enol tautomerization in the base mol­ecule.

## Synthesis and crystallization   

Single crystals of the title compounds (I)–(IV) were obtained by slow evaporation from aceto­nitrile solutions of 6-methyl­quinoline with chloro-nitro­benzoic acids in a 1:1 molar ratio at room temperature [80 ml aceto­nitrile solution of 6-methyl­quinoline (0.20 g) and chloro-nitro­benzoic acid (0.28 g for each acid)].

## Refinement   

Crystal data, data collection and structure refinement details are summarized in Table 5 ▸. All H atoms in compounds (I)–(IV) were found in difference-Fourier maps. The O-bound H atom in (II) was refined freely; the refined distance is given in Table 2 ▸. For (I), (III) and (IV), H atoms in the N⋯H⋯O hydrogen bonds were found to be disordered over two positions in difference-Fourier maps. Since the site-occupancy factors and isotropic displacement parameters are strongly collated, the positional parameters and occupancy factors were refined, with bond length restraints of N—H = 0.88 (1) Å and O—H = 0.84 (1) Å, and with *U*_iso_(H) = 1.5*U*_eq_(N or O); the refined distances are given in Tables 1 ▸, 3 ▸ and 4 ▸. Other H atoms were positioned geometrically (C—H = 0.95 Å) and treated as riding, with *U*_iso_(H) = 1.2 or 1.5*U*_eq_(C).

## Supplementary Material

Crystal structure: contains datablock(s) global, I, II, III, IV. DOI: 10.1107/S2056989020013134/hb7946sup1.cif

Structure factors: contains datablock(s) I. DOI: 10.1107/S2056989020013134/hb7946Isup2.hkl

Structure factors: contains datablock(s) II. DOI: 10.1107/S2056989020013134/hb7946IIsup3.hkl

Structure factors: contains datablock(s) III. DOI: 10.1107/S2056989020013134/hb7946IIIsup4.hkl

Structure factors: contains datablock(s) IV. DOI: 10.1107/S2056989020013134/hb7946IVsup5.hkl

Supporting information file. DOI: 10.1107/S2056989020013134/hb7946Isup6.cml

Supporting information file. DOI: 10.1107/S2056989020013134/hb7946IIsup7.cml

Supporting information file. DOI: 10.1107/S2056989020013134/hb7946IIIsup8.cml

Supporting information file. DOI: 10.1107/S2056989020013134/hb7946IVsup9.cml

CCDC references: 2034476, 2034475, 2034474, 2034473

Additional supporting information:  crystallographic information; 3D view; checkCIF report

## Figures and Tables

**Table 1:** Hydrogen-bond geometry (Å, °) for (I)

*D*—H⋯*A*	*D*—H	H⋯*A*	*D*⋯*A*	*D*—H⋯*A*
O1—H1⋯N2	0.85 (2)	1.70 (2)	2.5452 (12)	174 (3)
N2—H2⋯O1	0.88 (3)	1.66 (3)	2.5452 (12)	176 (3)
C8—H8⋯O4^i^	0.95	2.59	3.2307 (13)	125

Symmetry code: (i) 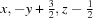.

**Table 2:** Hydrogen-bond geometry (Å, °) for (II)

*D*—H⋯*A*	*D*—H	H⋯*A*	*D*⋯*A*	*D*—H⋯*A*
O1—H1⋯N2	0.89 (2)	1.78 (2)	2.6569 (13)	169 (2)
C15—H15⋯O2	0.95	2.46	3.3211 (14)	151

**Table 3:** Hydrogen-bond geometry (Å, °) for (III)

*D*—H⋯*A*	*D*—H	H⋯*A*	*D*⋯*A*	*D*—H⋯*A*
O1—H1⋯N2	0.85 (3)	1.72 (3)	2.5640 (17)	174 (3)
N2—H2⋯O1	0.88 (4)	1.69 (4)	2.5640 (17)	170 (4)
C5—H5⋯.O2^i^	0.95	2.44	3.3245 (19)	155
C8—H8⋯.O2	0.95	2.46	3.1438 (19)	129

Symmetry code: (i) 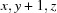.

**Table 4:** Hydrogen-bond geometry (Å, °) for (IV)

*D*—H⋯*A*	*D*—H	H⋯*A*	*D*⋯*A*	*D*—H⋯*A*
O1—H1⋯N2	0.84 (7)	1.70 (6)	2.514 (2)	163 (7)
N2—H2⋯O1	0.87 (4)	1.67 (5)	2.514 (2)	162 (4)
C10—H10⋯O2^i^	0.95	2.54	3.364 (3)	145

Symmetry code: (i) 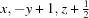.

**Table 5:** Experimental details

	(I)	(II)	(III)	(IV)
Crystal data				
Chemical formula	C_7_H_3.65_ClNO_4_·C_10_H_9.35_N	C_7_H_4_ClNO_4_·C_10_H_9_N	C_7_H_3.59_ClNO_4_·C_10_H_9.41_N	C_7_H_3.48_ClNO_4_·C_10_H_9.52_N
*M*_r_	344.74	344.74	344.75	344.75
Crystal system, space group	Monoclinic, *P*2_1_/*c*	Triclinic, *P* 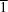	Orthorhombic, *P*2_1_2_1_2_1_	Monoclinic, *C* *c*
Temperature (K)	185	186	190	185
*a*, *b*, *c* (Å)	9.5055 (2), 8.3019 (4), 19.5865 (4)	6.8693 (3), 7.6482 (4), 15.1195 (4)	7.1156 (4), 7.5854 (4), 28.8599 (14)	7.4271 (6), 14.4348 (6), 16.2208 (7)
α, β, γ (°)	90, 95.7214 (7), 90	78.218 (3), 81.1923 (18), 77.754 (3)	90, 90, 90	90, 113.203 (3), 90
*V* (Å^3^)	1537.94 (8)	754.89 (6)	1557.70 (14)	1598.35 (16)
*Z*	4	2	4	4
Radiation type	Mo *K*α	Mo *K*α	Mo *K*α	Mo *K*α
μ (mm^−1^)	0.27	0.28	0.27	0.26
Crystal size (mm)	0.40 × 0.35 × 0.35	0.45 × 0.35 × 0.30	0.30 × 0.30 × 0.17	0.28 × 0.25 × 0.20

Data collection				
Diffractometer	Rigaku R-AXIS RAPIDII	Rigaku R-AXIS RAPIDII	Rigaku R-AXIS RAPIDII	Rigaku R-AXIS RAPIDII
Absorption correction	Numerical (*NUMABS*; Higashi, 1999 ▸)	Numerical (*NUMABS*; Higashi, 1999 ▸)	Numerical (*NUMABS*; Higashi, 1999 ▸)	Numerical (*NUMABS*; Higashi, 1999 ▸)
*T*_min_, *T*_max_	0.887, 0.909	0.891, 0.920	0.938, 0.955	0.931, 0.949
No. of measured, independent and observed [*I* > 2σ(*I*)] reflections	30539, 4487, 4065	15404, 4381, 3868	30061, 4532, 4365	16695, 4645, 4158
*R*_int_	0.025	0.023	0.017	0.015
(sin θ/λ)_max_ (Å^−1^)	0.704	0.703	0.703	0.703

Refinement				
*R*[*F* ^2^ > 2σ(*F* ^2^)], *wR*(*F* ^2^), *S*	0.034, 0.096, 1.06	0.036, 0.108, 1.05	0.028, 0.079, 1.06	0.030, 0.081, 1.09
No. of reflections	4487	4381	4532	4645
No. of parameters	225	222	225	225
No. of restraints	2	0	2	4
H-atom treatment	H atoms treated by a mixture of independent and constrained refinement	H atoms treated by a mixture of independent and constrained refinement	H atoms treated by a mixture of independent and constrained refinement	H atoms treated by a mixture of independent and constrained refinement
Δρ_max_, Δρ_min_ (e Å^−3^)	0.43, −0.22	0.48, −0.26	0.31, −0.26	0.35, −0.16
Absolute structure	–	–	Flack *x* determined using 1821 quotients [(*I* ^+^)−(*I* ^−^)]/[(*I* ^+^)+(*I* ^−^)] (Parsons *et al.*, 2013 ▸)	Flack *x* determined using 1899 quotients [(*I* ^+^)−(*I* ^−^)]/[(*I* ^+^)+(*I* ^−^)] (Parsons *et al.*, 2013 ▸)
Absolute structure parameter	–	–	−0.014 (8)	−0.023 (9)

Computer programs: *PROCESS-AUTO* (Rigaku, 2006 ▸), *SHELXT* (Sheldrick, 2015*a* ▸), *SHELXS97* (Sheldrick, 2008 ▸), *SHELXL* (Sheldrick, 2015*b* ▸), *ORTEP-3 for Windows* (Farrugia, 2012 ▸) and *Mercury* (Macrae *et al.*, 2020 ▸), *CrystalStructure* (Rigaku, 2018 ▸) and *PLATON* (Spek, 2020 ▸).

## supplementary crystallographic information

### 2-Chloro-4-nitrobenzoic acid–6-methylquinoline (1/1) (I) Crystal data

**Table:**

C_7_H_3.65_ClNO_4_·C_10_H_9.35_N	*F*(000) = 712.00
*M**_r_* = 344.74	*D*_x_ = 1.489 Mg m^−^^3^
Monoclinic, *P*2_1_/*c*	Mo *K*α radiation, λ = 0.71075 Å
*a* = 9.5055 (2) Å	Cell parameters from 26323 reflections
*b* = 8.3019 (4) Å	θ = 3.1–30.2°
*c* = 19.5865 (4) Å	µ = 0.27 mm^−^^1^
β = 95.7214 (7)°	*T* = 185 K
*V* = 1537.94 (8) Å^3^	Block, colorless
*Z* = 4	0.40 × 0.35 × 0.35 mm

### 2-Chloro-4-nitrobenzoic acid–6-methylquinoline (1/1) (I) Data collection

**Table:**

Rigaku R-AXIS RAPIDII diffractometer	4065 reflections with *I* > 2σ(*I*)
Detector resolution: 10.000 pixels mm^-1^	*R*_int_ = 0.025
ω scans	θ_max_ = 30.0°, θ_min_ = 3.2°
Absorption correction: numerical (*NUMABS*; Higashi, 1999)	*h* = −13→12
*T*_min_ = 0.887, *T*_max_ = 0.909	*k* = −11→11
30539 measured reflections	*l* = −27→26
4487 independent reflections	

### 2-Chloro-4-nitrobenzoic acid–6-methylquinoline (1/1) (I) Refinement

**Table:**

Refinement on *F*^2^	Secondary atom site location: difference Fourier map
*R*[*F*^2^ > 2σ(*F*^2^)] = 0.034	Hydrogen site location: mixed
*wR*(*F*^2^) = 0.096	H atoms treated by a mixture of independent and constrained refinement
*S* = 1.06	*w* = 1/[σ^2^(*F*_o_^2^) + (0.0572*P*)^2^ + 0.329*P*] where *P* = (*F*_o_^2^ + 2*F*_c_^2^)/3
4487 reflections	(Δ/σ)_max_ = 0.001
225 parameters	Δρ_max_ = 0.43 e Å^−^^3^
2 restraints	Δρ_min_ = −0.22 e Å^−^^3^
Primary atom site location: structure-invariant direct methods	

### 2-Chloro-4-nitrobenzoic acid–6-methylquinoline (1/1) (I) Special details

**Table:**

Geometry. All esds (except the esd in the dihedral angle between two l.s. planes) are estimated using the full covariance matrix. The cell esds are taken into account individually in the estimation of esds in distances, angles and torsion angles; correlations between esds in cell parameters are only used when they are defined by crystal symmetry. An approximate (isotropic) treatment of cell esds is used for estimating esds involving l.s. planes.

### 2-Chloro-4-nitrobenzoic acid–6-methylquinoline (1/1) (I) Fractional atomic coordinates and isotropic or equivalent isotropic displacement parameters (Å^2^)

**Table:**

	*x*	*y*	*z*	*U*_iso_*/*U*_eq_	Occ. (<1)
Cl1	0.87370 (3)	0.79426 (3)	0.44087 (2)	0.03134 (8)	
O1	0.42961 (8)	0.64577 (11)	0.42646 (5)	0.03635 (19)	
H1	0.401 (3)	0.576 (3)	0.3967 (11)	0.055*	0.65 (3)
O2	0.64551 (9)	0.55323 (11)	0.41332 (5)	0.0415 (2)	
O3	0.85703 (9)	1.17879 (11)	0.64471 (4)	0.03715 (19)	
O4	0.67137 (9)	1.12532 (11)	0.69548 (4)	0.03875 (19)	
N1	0.74667 (9)	1.10600 (10)	0.64913 (4)	0.02564 (17)	
N2	0.32928 (9)	0.44919 (10)	0.33444 (4)	0.02445 (16)	
H2	0.364 (4)	0.521 (4)	0.3649 (17)	0.037*	0.35 (3)
C1	0.61850 (9)	0.76377 (11)	0.49454 (4)	0.02042 (16)	
C2	0.75219 (9)	0.83696 (11)	0.49820 (4)	0.02126 (17)	
C3	0.79336 (9)	0.95089 (11)	0.54813 (5)	0.02240 (17)	
H3	0.883075	1.001906	0.549641	0.027*	
C4	0.70028 (10)	0.98778 (11)	0.59541 (4)	0.02162 (17)	
C5	0.56791 (10)	0.91744 (12)	0.59456 (5)	0.02394 (18)	
H5	0.506368	0.944280	0.628184	0.029*	
C6	0.52786 (10)	0.80690 (11)	0.54331 (5)	0.02350 (18)	
H6	0.436619	0.759387	0.541313	0.028*	
C7	0.56641 (10)	0.64206 (11)	0.44026 (5)	0.02361 (17)	
C8	0.40800 (11)	0.39905 (13)	0.28726 (5)	0.0284 (2)	
H8	0.500827	0.441308	0.287010	0.034*	
C9	0.36003 (12)	0.28509 (13)	0.23709 (5)	0.0314 (2)	
H9	0.418985	0.252719	0.203149	0.038*	
C10	0.22700 (12)	0.22130 (12)	0.23779 (5)	0.0291 (2)	
H10	0.193332	0.143923	0.204340	0.035*	
C11	0.14049 (10)	0.27092 (11)	0.28827 (5)	0.02387 (18)	
C12	0.00279 (11)	0.20841 (12)	0.29329 (5)	0.0285 (2)	
H12	−0.034075	0.128787	0.261562	0.034*	
C13	−0.07753 (11)	0.26074 (13)	0.34293 (6)	0.0294 (2)	
C14	−0.02173 (11)	0.38120 (13)	0.38945 (6)	0.0299 (2)	
H14	−0.078279	0.419813	0.423309	0.036*	
C15	0.11154 (11)	0.44368 (12)	0.38702 (5)	0.02705 (19)	
H15	0.146830	0.523118	0.419192	0.032*	
C16	0.19535 (10)	0.38878 (11)	0.33634 (5)	0.02220 (17)	
C17	−0.22358 (12)	0.19441 (16)	0.34909 (8)	0.0408 (3)	
H17A	−0.290638	0.283656	0.350772	0.061*	
H17B	−0.222599	0.130421	0.391184	0.061*	
H17C	−0.252335	0.126106	0.309332	0.061*	

### 2-Chloro-4-nitrobenzoic acid–6-methylquinoline (1/1) (I) Atomic displacement parameters (Å^2^)

**Table:**

	*U*^11^	*U*^22^	*U*^33^	*U*^12^	*U*^13^	*U*^23^
Cl1	0.02668 (13)	0.03764 (15)	0.03157 (13)	−0.00822 (9)	0.01214 (9)	−0.01037 (9)
O1	0.0247 (4)	0.0378 (4)	0.0445 (4)	0.0002 (3)	−0.0067 (3)	−0.0172 (3)
O2	0.0293 (4)	0.0412 (5)	0.0547 (5)	−0.0052 (3)	0.0083 (3)	−0.0239 (4)
O3	0.0343 (4)	0.0387 (4)	0.0387 (4)	−0.0130 (3)	0.0050 (3)	−0.0133 (3)
O4	0.0452 (5)	0.0433 (5)	0.0297 (4)	−0.0057 (4)	0.0133 (3)	−0.0115 (3)
N1	0.0282 (4)	0.0247 (4)	0.0239 (4)	−0.0008 (3)	0.0022 (3)	−0.0028 (3)
N2	0.0244 (4)	0.0227 (4)	0.0254 (4)	−0.0023 (3)	−0.0015 (3)	−0.0010 (3)
C1	0.0196 (4)	0.0194 (4)	0.0219 (4)	−0.0016 (3)	0.0006 (3)	0.0011 (3)
C2	0.0195 (4)	0.0228 (4)	0.0219 (4)	−0.0016 (3)	0.0043 (3)	−0.0005 (3)
C3	0.0197 (4)	0.0235 (4)	0.0241 (4)	−0.0042 (3)	0.0024 (3)	−0.0010 (3)
C4	0.0235 (4)	0.0200 (4)	0.0211 (4)	−0.0012 (3)	0.0011 (3)	−0.0012 (3)
C5	0.0219 (4)	0.0258 (4)	0.0247 (4)	−0.0006 (3)	0.0055 (3)	−0.0004 (3)
C6	0.0193 (4)	0.0246 (4)	0.0268 (4)	−0.0033 (3)	0.0034 (3)	0.0000 (3)
C7	0.0247 (4)	0.0213 (4)	0.0247 (4)	−0.0042 (3)	0.0019 (3)	−0.0004 (3)
C8	0.0274 (4)	0.0280 (5)	0.0297 (5)	−0.0020 (4)	0.0029 (4)	0.0016 (4)
C9	0.0375 (5)	0.0320 (5)	0.0256 (4)	0.0002 (4)	0.0072 (4)	−0.0014 (4)
C10	0.0389 (5)	0.0267 (5)	0.0212 (4)	−0.0027 (4)	0.0000 (4)	−0.0031 (3)
C11	0.0276 (4)	0.0214 (4)	0.0214 (4)	−0.0014 (3)	−0.0038 (3)	0.0008 (3)
C12	0.0286 (5)	0.0257 (4)	0.0295 (5)	−0.0047 (3)	−0.0060 (4)	0.0000 (3)
C13	0.0238 (4)	0.0268 (4)	0.0366 (5)	−0.0010 (4)	−0.0017 (4)	0.0065 (4)
C14	0.0280 (5)	0.0274 (5)	0.0346 (5)	0.0035 (4)	0.0052 (4)	0.0015 (4)
C15	0.0292 (5)	0.0234 (4)	0.0282 (4)	0.0006 (3)	0.0011 (3)	−0.0032 (3)
C16	0.0237 (4)	0.0197 (4)	0.0223 (4)	−0.0004 (3)	−0.0023 (3)	0.0003 (3)
C17	0.0269 (5)	0.0399 (6)	0.0553 (7)	−0.0065 (4)	0.0026 (5)	0.0073 (5)

### 2-Chloro-4-nitrobenzoic acid–6-methylquinoline (1/1) (I) Geometric parameters (Å, º)

**Table:**

Cl1—C2	1.7262 (9)	C8—C9	1.4069 (14)
O1—C7	1.3019 (12)	C8—H8	0.9500
O1—H1	0.847 (10)	C9—C10	1.3722 (16)
O2—C7	1.2111 (13)	C9—H9	0.9500
O3—N1	1.2211 (12)	C10—C11	1.4091 (14)
O4—N1	1.2215 (11)	C10—H10	0.9500
N1—C4	1.4739 (12)	C11—C12	1.4206 (14)
N2—C8	1.3134 (13)	C11—C16	1.4205 (12)
N2—C16	1.3722 (12)	C12—C13	1.3656 (16)
N2—H2	0.883 (10)	C12—H12	0.9500
C1—C6	1.3954 (13)	C13—C14	1.4196 (15)
C1—C2	1.4039 (12)	C13—C17	1.5096 (15)
C1—C7	1.5137 (12)	C14—C15	1.3741 (15)
C2—C3	1.3884 (12)	C14—H14	0.9500
C3—C4	1.3774 (13)	C15—C16	1.4090 (14)
C3—H3	0.9500	C15—H15	0.9500
C4—C5	1.3857 (13)	C17—H17A	0.9800
C5—C6	1.3851 (13)	C17—H17B	0.9800
C5—H5	0.9500	C17—H17C	0.9800
C6—H6	0.9500		
			
C7—O1—H1	112.0 (19)	C10—C9—C8	119.17 (10)
O3—N1—O4	123.98 (9)	C10—C9—H9	120.4
O3—N1—C4	118.49 (8)	C8—C9—H9	120.4
O4—N1—C4	117.53 (8)	C9—C10—C11	119.80 (9)
C8—N2—C16	119.89 (8)	C9—C10—H10	120.1
C8—N2—H2	119 (3)	C11—C10—H10	120.1
C16—N2—H2	121 (3)	C10—C11—C12	123.23 (9)
C6—C1—C2	118.13 (8)	C10—C11—C16	117.72 (9)
C6—C1—C7	118.10 (8)	C12—C11—C16	119.05 (9)
C2—C1—C7	123.77 (8)	C13—C12—C11	121.16 (9)
C3—C2—C1	121.40 (8)	C13—C12—H12	119.4
C3—C2—Cl1	115.99 (7)	C11—C12—H12	119.4
C1—C2—Cl1	122.60 (7)	C12—C13—C14	118.79 (9)
C4—C3—C2	118.04 (8)	C12—C13—C17	121.65 (10)
C4—C3—H3	121.0	C14—C13—C17	119.56 (10)
C2—C3—H3	121.0	C15—C14—C13	122.03 (10)
C3—C4—C5	122.79 (8)	C15—C14—H14	119.0
C3—C4—N1	117.48 (8)	C13—C14—H14	119.0
C5—C4—N1	119.73 (8)	C14—C15—C16	119.36 (9)
C6—C5—C4	118.14 (8)	C14—C15—H15	120.3
C6—C5—H5	120.9	C16—C15—H15	120.3
C4—C5—H5	120.9	N2—C16—C15	119.48 (8)
C5—C6—C1	121.48 (8)	N2—C16—C11	120.93 (9)
C5—C6—H6	119.3	C15—C16—C11	119.59 (9)
C1—C6—H6	119.3	C13—C17—H17A	109.5
O2—C7—O1	125.07 (9)	C13—C17—H17B	109.5
O2—C7—C1	122.59 (9)	H17A—C17—H17B	109.5
O1—C7—C1	112.34 (8)	C13—C17—H17C	109.5
N2—C8—C9	122.47 (9)	H17A—C17—H17C	109.5
N2—C8—H8	118.8	H17B—C17—H17C	109.5
C9—C8—H8	118.8		
			
C6—C1—C2—C3	−0.91 (13)	C16—N2—C8—C9	0.66 (15)
C7—C1—C2—C3	178.41 (8)	N2—C8—C9—C10	−1.13 (16)
C6—C1—C2—Cl1	−179.86 (7)	C8—C9—C10—C11	0.25 (16)
C7—C1—C2—Cl1	−0.55 (13)	C9—C10—C11—C12	−178.73 (9)
C1—C2—C3—C4	1.56 (14)	C9—C10—C11—C16	0.97 (14)
Cl1—C2—C3—C4	−179.42 (7)	C10—C11—C12—C13	−179.69 (9)
C2—C3—C4—C5	−0.70 (14)	C16—C11—C12—C13	0.62 (14)
C2—C3—C4—N1	178.55 (8)	C11—C12—C13—C14	0.77 (15)
O3—N1—C4—C3	8.31 (13)	C11—C12—C13—C17	−179.57 (9)
O4—N1—C4—C3	−171.89 (9)	C12—C13—C14—C15	−1.57 (16)
O3—N1—C4—C5	−172.41 (9)	C17—C13—C14—C15	178.76 (10)
O4—N1—C4—C5	7.39 (13)	C13—C14—C15—C16	0.91 (15)
C3—C4—C5—C6	−0.78 (14)	C8—N2—C16—C15	−179.81 (9)
N1—C4—C5—C6	179.98 (8)	C8—N2—C16—C11	0.65 (14)
C4—C5—C6—C1	1.46 (14)	C14—C15—C16—N2	−179.02 (9)
C2—C1—C6—C5	−0.64 (14)	C14—C15—C16—C11	0.53 (14)
C7—C1—C6—C5	180.00 (8)	C10—C11—C16—N2	−1.45 (13)
C6—C1—C7—O2	−151.53 (10)	C12—C11—C16—N2	178.26 (9)
C2—C1—C7—O2	29.15 (15)	C10—C11—C16—C15	179.01 (9)
C6—C1—C7—O1	28.79 (12)	C12—C11—C16—C15	−1.28 (13)
C2—C1—C7—O1	−150.52 (9)		

### 2-Chloro-4-nitrobenzoic acid–6-methylquinoline (1/1) (I) Hydrogen-bond geometry (Å, º)

**Table:**

*D*—H···*A*	*D*—H	H···*A*	*D*···*A*	*D*—H···*A*
O1—H1···N2	0.85 (2)	1.70 (2)	2.5452 (12)	174 (3)
N2—H2···O1	0.88 (3)	1.66 (3)	2.5452 (12)	176 (3)
C8—H8···O4^i^	0.95	2.59	3.2307 (13)	125

Symmetry code: (i) *x*, −*y*+3/2, *z*−1/2.

### 2-Chloro-5-nitrobenzoic acid–6-methylquinoline (1/1) (II) Crystal data

**Table:**

C_7_H_4_ClNO_4_·C_10_H_9_N	*Z* = 2
*M**_r_* = 344.74	*F*(000) = 356.00
Triclinic, *P*1	*D*_x_ = 1.517 Mg m^−^^3^
*a* = 6.8693 (3) Å	Mo *K*α radiation, λ = 0.71075 Å
*b* = 7.6482 (4) Å	Cell parameters from 13517 reflections
*c* = 15.1195 (4) Å	θ = 3.1–30.1°
α = 78.218 (3)°	µ = 0.28 mm^−^^1^
β = 81.1923 (18)°	*T* = 186 K
γ = 77.754 (3)°	Block, colorless
*V* = 754.89 (6) Å^3^	0.45 × 0.35 × 0.30 mm

### 2-Chloro-5-nitrobenzoic acid–6-methylquinoline (1/1) (II) Data collection

**Table:**

Rigaku R-AXIS RAPIDII diffractometer	3868 reflections with *I* > 2σ(*I*)
Detector resolution: 10.000 pixels mm^-1^	*R*_int_ = 0.023
ω scans	θ_max_ = 30.0°, θ_min_ = 3.1°
Absorption correction: numerical (*NUMABS*; Higashi, 1999)	*h* = −9→9
*T*_min_ = 0.891, *T*_max_ = 0.920	*k* = −10→10
15404 measured reflections	*l* = −20→21
4381 independent reflections	

### 2-Chloro-5-nitrobenzoic acid–6-methylquinoline (1/1) (II) Refinement

**Table:**

Refinement on *F*^2^	Primary atom site location: structure-invariant direct methods
Least-squares matrix: full	Secondary atom site location: difference Fourier map
*R*[*F*^2^ > 2σ(*F*^2^)] = 0.036	Hydrogen site location: mixed
*wR*(*F*^2^) = 0.108	H atoms treated by a mixture of independent and constrained refinement
*S* = 1.05	*w* = 1/[σ^2^(*F*_o_^2^) + (0.0662*P*)^2^ + 0.1585*P*] where *P* = (*F*_o_^2^ + 2*F*_c_^2^)/3
4381 reflections	(Δ/σ)_max_ = 0.001
222 parameters	Δρ_max_ = 0.48 e Å^−^^3^
0 restraints	Δρ_min_ = −0.26 e Å^−^^3^

### 2-Chloro-5-nitrobenzoic acid–6-methylquinoline (1/1) (II) Special details

**Table:**

Geometry. All esds (except the esd in the dihedral angle between two l.s. planes) are estimated using the full covariance matrix. The cell esds are taken into account individually in the estimation of esds in distances, angles and torsion angles; correlations between esds in cell parameters are only used when they are defined by crystal symmetry. An approximate (isotropic) treatment of cell esds is used for estimating esds involving l.s. planes.

### 2-Chloro-5-nitrobenzoic acid–6-methylquinoline (1/1) (II) Fractional atomic coordinates and isotropic or equivalent isotropic displacement parameters (Å^2^)

**Table:**

	*x*	*y*	*z*	*U*_iso_*/*U*_eq_	
Cl1	0.11678 (4)	0.98086 (4)	0.61864 (2)	0.03561 (10)	
O1	0.21038 (15)	0.43541 (12)	0.54709 (6)	0.0366 (2)	
O2	0.28610 (18)	0.71213 (14)	0.49895 (6)	0.0478 (3)	
O3	0.18489 (15)	0.29378 (13)	0.96192 (6)	0.0392 (2)	
O4	0.25163 (16)	0.14131 (12)	0.85221 (6)	0.0405 (2)	
N1	0.20962 (14)	0.28451 (13)	0.88057 (6)	0.02681 (18)	
N2	0.29763 (14)	0.34416 (12)	0.38322 (6)	0.02569 (18)	
C1	0.19890 (14)	0.60827 (13)	0.65842 (7)	0.02253 (19)	
C2	0.15118 (15)	0.77299 (14)	0.69035 (7)	0.02393 (19)	
C3	0.12289 (16)	0.77660 (14)	0.78333 (7)	0.0264 (2)	
H3	0.091426	0.890115	0.803551	0.032*	
C4	0.14004 (15)	0.61713 (14)	0.84642 (7)	0.0254 (2)	
H4	0.119312	0.619066	0.909822	0.030*	
C5	0.18819 (14)	0.45521 (13)	0.81441 (6)	0.02191 (18)	
C6	0.21638 (15)	0.44738 (13)	0.72259 (7)	0.02259 (19)	
H6	0.247515	0.333038	0.703216	0.027*	
C7	0.23535 (16)	0.59389 (15)	0.55899 (7)	0.0274 (2)	
C8	0.34550 (16)	0.16678 (15)	0.38795 (7)	0.0279 (2)	
H8	0.346849	0.091817	0.446377	0.033*	
C9	0.39475 (17)	0.08253 (14)	0.31103 (8)	0.0284 (2)	
H9	0.429397	−0.046122	0.317664	0.034*	
C10	0.39200 (16)	0.18891 (14)	0.22624 (7)	0.0267 (2)	
H10	0.424134	0.134301	0.173486	0.032*	
C11	0.34115 (14)	0.38015 (13)	0.21768 (7)	0.02121 (18)	
C12	0.33604 (15)	0.50006 (14)	0.13253 (7)	0.02423 (19)	
H12	0.364652	0.451051	0.078062	0.029*	
C13	0.29069 (15)	0.68496 (14)	0.12730 (7)	0.02410 (19)	
C14	0.24533 (16)	0.75428 (14)	0.20953 (8)	0.0271 (2)	
H14	0.212931	0.882237	0.206693	0.032*	
C15	0.24652 (16)	0.64331 (14)	0.29307 (7)	0.0264 (2)	
H15	0.214505	0.694420	0.346914	0.032*	
C16	0.29544 (14)	0.45298 (13)	0.29885 (6)	0.02121 (18)	
C17	0.28690 (19)	0.81553 (17)	0.03801 (8)	0.0341 (2)	
H17A	0.323370	0.747133	−0.012173	0.051*	
H17B	0.382979	0.895687	0.034211	0.051*	
H17C	0.151890	0.888699	0.033913	0.051*	
H1	0.243 (3)	0.420 (3)	0.4899 (15)	0.064 (6)*	

### 2-Chloro-5-nitrobenzoic acid–6-methylquinoline (1/1) (II) Atomic displacement parameters (Å^2^)

**Table:**

	*U*^11^	*U*^22^	*U*^33^	*U*^12^	*U*^13^	*U*^23^
Cl1	0.03973 (16)	0.02267 (14)	0.03659 (16)	−0.00175 (10)	−0.00100 (11)	0.00595 (10)
O1	0.0568 (6)	0.0320 (4)	0.0209 (4)	−0.0110 (4)	0.0014 (3)	−0.0059 (3)
O2	0.0815 (8)	0.0429 (5)	0.0220 (4)	−0.0271 (5)	−0.0024 (4)	0.0010 (4)
O3	0.0584 (6)	0.0364 (5)	0.0194 (4)	−0.0069 (4)	−0.0037 (3)	0.0005 (3)
O4	0.0648 (6)	0.0216 (4)	0.0315 (4)	−0.0031 (4)	−0.0064 (4)	−0.0010 (3)
N1	0.0315 (4)	0.0243 (4)	0.0227 (4)	−0.0046 (3)	−0.0038 (3)	0.0002 (3)
N2	0.0299 (4)	0.0250 (4)	0.0208 (4)	−0.0049 (3)	−0.0029 (3)	−0.0012 (3)
C1	0.0227 (4)	0.0234 (5)	0.0203 (4)	−0.0042 (3)	−0.0029 (3)	−0.0012 (3)
C2	0.0222 (4)	0.0215 (4)	0.0262 (5)	−0.0043 (3)	−0.0032 (3)	0.0007 (3)
C3	0.0291 (5)	0.0209 (4)	0.0292 (5)	−0.0040 (4)	−0.0023 (4)	−0.0061 (4)
C4	0.0278 (5)	0.0265 (5)	0.0221 (4)	−0.0054 (4)	−0.0022 (3)	−0.0050 (4)
C5	0.0233 (4)	0.0213 (4)	0.0202 (4)	−0.0048 (3)	−0.0034 (3)	−0.0001 (3)
C6	0.0250 (4)	0.0213 (4)	0.0208 (4)	−0.0038 (3)	−0.0027 (3)	−0.0028 (3)
C7	0.0303 (5)	0.0297 (5)	0.0212 (5)	−0.0052 (4)	−0.0042 (4)	−0.0016 (4)
C8	0.0298 (5)	0.0249 (5)	0.0269 (5)	−0.0066 (4)	−0.0042 (4)	0.0027 (4)
C9	0.0310 (5)	0.0183 (4)	0.0347 (5)	−0.0046 (4)	−0.0040 (4)	−0.0015 (4)
C10	0.0308 (5)	0.0201 (4)	0.0293 (5)	−0.0050 (4)	−0.0006 (4)	−0.0065 (4)
C11	0.0217 (4)	0.0193 (4)	0.0226 (4)	−0.0049 (3)	−0.0014 (3)	−0.0035 (3)
C12	0.0274 (5)	0.0250 (5)	0.0203 (4)	−0.0060 (4)	−0.0013 (3)	−0.0039 (3)
C13	0.0252 (4)	0.0234 (5)	0.0230 (5)	−0.0067 (4)	−0.0047 (3)	0.0010 (3)
C14	0.0326 (5)	0.0188 (4)	0.0298 (5)	−0.0035 (4)	−0.0067 (4)	−0.0033 (4)
C15	0.0335 (5)	0.0209 (5)	0.0249 (5)	−0.0023 (4)	−0.0043 (4)	−0.0064 (4)
C16	0.0223 (4)	0.0204 (4)	0.0206 (4)	−0.0042 (3)	−0.0024 (3)	−0.0028 (3)
C17	0.0424 (6)	0.0308 (5)	0.0268 (5)	−0.0099 (5)	−0.0071 (4)	0.0059 (4)

### 2-Chloro-5-nitrobenzoic acid–6-methylquinoline (1/1) (II) Geometric parameters (Å, º)

**Table:**

Cl1—C2	1.7245 (10)	C8—C9	1.4055 (16)
O1—C7	1.3106 (14)	C8—H8	0.9500
O1—H1	0.89 (2)	C9—C10	1.3713 (15)
O2—C7	1.2104 (14)	C9—H9	0.9500
O3—N1	1.2302 (12)	C10—C11	1.4134 (13)
O4—N1	1.2192 (13)	C10—H10	0.9500
N1—C5	1.4686 (13)	C11—C16	1.4149 (13)
N2—C8	1.3161 (14)	C11—C12	1.4205 (13)
N2—C16	1.3737 (12)	C12—C13	1.3705 (14)
C1—C2	1.3972 (14)	C12—H12	0.9500
C1—C6	1.3984 (13)	C13—C14	1.4157 (15)
C1—C7	1.5084 (14)	C13—C17	1.5066 (14)
C2—C3	1.3946 (15)	C14—C15	1.3706 (15)
C3—C4	1.3819 (15)	C14—H14	0.9500
C3—H3	0.9500	C15—C16	1.4103 (14)
C4—C5	1.3781 (14)	C15—H15	0.9500
C4—H4	0.9500	C17—H17A	0.9800
C5—C6	1.3838 (14)	C17—H17B	0.9800
C6—H6	0.9500	C17—H17C	0.9800
			
C7—O1—H1	112.6 (13)	C10—C9—H9	120.5
O4—N1—O3	123.42 (10)	C8—C9—H9	120.5
O4—N1—C5	118.51 (9)	C9—C10—C11	119.73 (10)
O3—N1—C5	118.07 (9)	C9—C10—H10	120.1
C8—N2—C16	118.47 (9)	C11—C10—H10	120.1
C2—C1—C6	117.92 (9)	C10—C11—C16	117.36 (9)
C2—C1—C7	123.90 (9)	C10—C11—C12	123.30 (9)
C6—C1—C7	118.17 (9)	C16—C11—C12	119.33 (9)
C3—C2—C1	120.97 (9)	C13—C12—C11	121.38 (9)
C3—C2—Cl1	116.41 (8)	C13—C12—H12	119.3
C1—C2—Cl1	122.59 (8)	C11—C12—H12	119.3
C4—C3—C2	120.83 (10)	C12—C13—C14	118.14 (9)
C4—C3—H3	119.6	C12—C13—C17	122.58 (10)
C2—C3—H3	119.6	C14—C13—C17	119.27 (10)
C5—C4—C3	117.86 (9)	C15—C14—C13	122.32 (9)
C5—C4—H4	121.1	C15—C14—H14	118.8
C3—C4—H4	121.1	C13—C14—H14	118.8
C4—C5—C6	122.59 (9)	C14—C15—C16	119.78 (9)
C4—C5—N1	118.54 (9)	C14—C15—H15	120.1
C6—C5—N1	118.86 (9)	C16—C15—H15	120.1
C5—C6—C1	119.81 (9)	N2—C16—C15	118.88 (9)
C5—C6—H6	120.1	N2—C16—C11	122.08 (9)
C1—C6—H6	120.1	C15—C16—C11	119.03 (9)
O2—C7—O1	124.98 (10)	C13—C17—H17A	109.5
O2—C7—C1	123.97 (10)	C13—C17—H17B	109.5
O1—C7—C1	111.02 (9)	H17A—C17—H17B	109.5
N2—C8—C9	123.38 (10)	C13—C17—H17C	109.5
N2—C8—H8	118.3	H17A—C17—H17C	109.5
C9—C8—H8	118.3	H17B—C17—H17C	109.5
C10—C9—C8	118.97 (10)		
			
C6—C1—C2—C3	0.31 (15)	C16—N2—C8—C9	0.03 (16)
C7—C1—C2—C3	−178.82 (10)	N2—C8—C9—C10	−0.48 (17)
C6—C1—C2—Cl1	−177.73 (7)	C8—C9—C10—C11	0.35 (16)
C7—C1—C2—Cl1	3.14 (14)	C9—C10—C11—C16	0.19 (15)
C1—C2—C3—C4	−0.43 (16)	C9—C10—C11—C12	179.57 (10)
Cl1—C2—C3—C4	177.73 (8)	C10—C11—C12—C13	−178.37 (10)
C2—C3—C4—C5	0.75 (16)	C16—C11—C12—C13	0.99 (15)
C3—C4—C5—C6	−1.02 (16)	C11—C12—C13—C14	−1.05 (15)
C3—C4—C5—N1	179.20 (9)	C11—C12—C13—C17	179.13 (9)
O4—N1—C5—C4	179.79 (10)	C12—C13—C14—C15	0.36 (16)
O3—N1—C5—C4	−0.50 (15)	C17—C13—C14—C15	−179.82 (10)
O4—N1—C5—C6	−0.01 (15)	C13—C14—C15—C16	0.40 (16)
O3—N1—C5—C6	179.71 (10)	C8—N2—C16—C15	−179.29 (10)
C4—C5—C6—C1	0.93 (16)	C8—N2—C16—C11	0.55 (15)
N1—C5—C6—C1	−179.29 (8)	C14—C15—C16—N2	179.39 (9)
C2—C1—C6—C5	−0.54 (15)	C14—C15—C16—C11	−0.45 (15)
C7—C1—C6—C5	178.64 (9)	C10—C11—C16—N2	−0.65 (14)
C2—C1—C7—O2	23.08 (17)	C12—C11—C16—N2	179.94 (9)
C6—C1—C7—O2	−156.05 (12)	C10—C11—C16—C15	179.19 (9)
C2—C1—C7—O1	−158.73 (10)	C12—C11—C16—C15	−0.22 (14)
C6—C1—C7—O1	22.14 (13)		

### 2-Chloro-5-nitrobenzoic acid–6-methylquinoline (1/1) (II) Hydrogen-bond geometry (Å, º)

**Table:**

*D*—H···*A*	*D*—H	H···*A*	*D*···*A*	*D*—H···*A*
O1—H1···N2	0.89 (2)	1.78 (2)	2.6569 (13)	169 (2)
C15—H15···O2	0.95	2.46	3.3211 (14)	151

### 3-Chloro-2-nitrobenzoic acid–6-methylquinoline (1/1) (III) Crystal data

**Table:**

C_7_H_3.59_ClNO_4_·C_10_H_9.41_N	*D*_x_ = 1.470 Mg m^−^^3^
*M**_r_* = 344.75	Mo *K*α radiation, λ = 0.71075 Å
Orthorhombic, *P*2_1_2_1_2_1_	Cell parameters from 28109 reflections
*a* = 7.1156 (4) Å	θ = 3.0–30.0°
*b* = 7.5854 (4) Å	µ = 0.27 mm^−^^1^
*c* = 28.8599 (14) Å	*T* = 190 K
*V* = 1557.70 (14) Å^3^	Block, colorless
*Z* = 4	0.30 × 0.30 × 0.17 mm
*F*(000) = 712.00	

### 3-Chloro-2-nitrobenzoic acid–6-methylquinoline (1/1) (III) Data collection

**Table:**

Rigaku R-AXIS RAPIDII diffractometer	4365 reflections with *I* > 2σ(*I*)
Detector resolution: 10.000 pixels mm^-1^	*R*_int_ = 0.017
ω scans	θ_max_ = 30.0°, θ_min_ = 3.0°
Absorption correction: numerical (*NUMABS*; Higashi, 1999)	*h* = −10→9
*T*_min_ = 0.938, *T*_max_ = 0.955	*k* = −10→10
30061 measured reflections	*l* = −39→40
4532 independent reflections	

### 3-Chloro-2-nitrobenzoic acid–6-methylquinoline (1/1) (III) Refinement

**Table:**

Refinement on *F*^2^	Secondary atom site location: difference Fourier map
Least-squares matrix: full	Hydrogen site location: inferred from neighbouring sites
*R*[*F*^2^ > 2σ(*F*^2^)] = 0.028	H atoms treated by a mixture of independent and constrained refinement
*wR*(*F*^2^) = 0.079	*w* = 1/[σ^2^(*F*_o_^2^) + (0.0546*P*)^2^ + 0.1455*P*] where *P* = (*F*_o_^2^ + 2*F*_c_^2^)/3
*S* = 1.06	(Δ/σ)_max_ < 0.001
4532 reflections	Δρ_max_ = 0.31 e Å^−^^3^
225 parameters	Δρ_min_ = −0.26 e Å^−^^3^
2 restraints	Absolute structure: Flack *x* determined using 1821 quotients [(*I*^+^)-(*I*^-^)]/[(*I*^+^)+(*I*^-^)] (Parsons *et al.*, 2013)
Primary atom site location: structure-invariant direct methods	Absolute structure parameter: −0.014 (8)

### 3-Chloro-2-nitrobenzoic acid–6-methylquinoline (1/1) (III) Special details

**Table:**

Geometry. All esds (except the esd in the dihedral angle between two l.s. planes) are estimated using the full covariance matrix. The cell esds are taken into account individually in the estimation of esds in distances, angles and torsion angles; correlations between esds in cell parameters are only used when they are defined by crystal symmetry. An approximate (isotropic) treatment of cell esds is used for estimating esds involving l.s. planes.

### 3-Chloro-2-nitrobenzoic acid–6-methylquinoline (1/1) (III) Fractional atomic coordinates and isotropic or equivalent isotropic displacement parameters (Å^2^)

**Table:**

	*x*	*y*	*z*	*U*_iso_*/*U*_eq_	Occ. (<1)
Cl1	0.53139 (7)	0.88067 (5)	0.01741 (2)	0.03788 (11)	
O1	0.27295 (18)	0.75649 (15)	0.22566 (4)	0.0324 (2)	
H1	0.236 (6)	0.671 (4)	0.2421 (12)	0.049*	0.59 (4)
O2	0.3168 (2)	0.54391 (16)	0.17307 (4)	0.0419 (3)	
O3	0.2617 (2)	0.56590 (17)	0.07241 (4)	0.0410 (3)	
O4	0.55968 (19)	0.53587 (15)	0.08592 (5)	0.0385 (3)	
N1	0.41299 (18)	0.61877 (16)	0.08702 (4)	0.0263 (2)	
N2	0.18381 (18)	0.49896 (17)	0.27884 (4)	0.0260 (2)	
H2	0.203 (8)	0.585 (5)	0.2590 (16)	0.039*	0.41 (4)
C1	0.37616 (18)	0.84027 (16)	0.15112 (4)	0.0210 (2)	
C2	0.42073 (19)	0.80125 (17)	0.10505 (4)	0.0216 (2)	
C3	0.4747 (2)	0.93226 (17)	0.07416 (4)	0.0240 (2)	
C4	0.4842 (2)	1.10660 (18)	0.08878 (5)	0.0265 (3)	
H4	0.520281	1.196857	0.067766	0.032*	
C5	0.4404 (2)	1.14758 (18)	0.13447 (5)	0.0272 (3)	
H5	0.446936	1.266384	0.144760	0.033*	
C6	0.38715 (19)	1.01549 (18)	0.16517 (5)	0.0246 (2)	
H6	0.357703	1.045362	0.196302	0.030*	
C7	0.3188 (2)	0.69810 (18)	0.18471 (5)	0.0245 (3)	
C8	0.2211 (2)	0.3372 (2)	0.26449 (5)	0.0297 (3)	
H8	0.261320	0.320660	0.233404	0.036*	
C9	0.2039 (2)	0.18918 (19)	0.29319 (5)	0.0307 (3)	
H9	0.229500	0.074532	0.281546	0.037*	
C10	0.1493 (2)	0.21234 (19)	0.33840 (5)	0.0282 (3)	
H10	0.138409	0.113530	0.358444	0.034*	
C11	0.10940 (18)	0.38383 (19)	0.35510 (4)	0.0234 (2)	
C12	0.0566 (2)	0.4191 (2)	0.40170 (5)	0.0269 (3)	
H12	0.046576	0.324225	0.423028	0.032*	
C13	0.0200 (2)	0.5876 (2)	0.41648 (5)	0.0283 (3)	
C14	0.0319 (2)	0.7280 (2)	0.38397 (5)	0.0311 (3)	
H14	0.003183	0.844465	0.393774	0.037*	
C15	0.0841 (2)	0.69943 (19)	0.33867 (5)	0.0292 (3)	
H15	0.091762	0.795472	0.317624	0.035*	
C16	0.12609 (19)	0.52672 (18)	0.32360 (5)	0.0233 (2)	
C17	−0.0287 (3)	0.6272 (3)	0.46621 (5)	0.0399 (4)	
H17A	−0.128360	0.716224	0.467272	0.060*	
H17B	0.082912	0.671801	0.482279	0.060*	
H17C	−0.072283	0.519138	0.481428	0.060*	

### 3-Chloro-2-nitrobenzoic acid–6-methylquinoline (1/1) (III) Atomic displacement parameters (Å^2^)

**Table:**

	*U*^11^	*U*^22^	*U*^33^	*U*^12^	*U*^13^	*U*^23^
Cl1	0.0610 (2)	0.03165 (18)	0.02096 (15)	−0.00237 (17)	0.01009 (15)	0.00035 (13)
O1	0.0498 (7)	0.0250 (5)	0.0223 (5)	−0.0041 (5)	0.0099 (5)	−0.0005 (4)
O2	0.0751 (9)	0.0223 (5)	0.0282 (5)	−0.0037 (5)	0.0160 (6)	0.0008 (4)
O3	0.0507 (7)	0.0332 (6)	0.0390 (6)	−0.0137 (5)	−0.0115 (5)	−0.0032 (5)
O4	0.0478 (6)	0.0249 (5)	0.0427 (6)	0.0058 (5)	0.0072 (5)	−0.0060 (5)
N1	0.0391 (6)	0.0201 (5)	0.0197 (5)	−0.0037 (5)	0.0015 (4)	−0.0007 (4)
N2	0.0325 (6)	0.0245 (5)	0.0209 (5)	−0.0020 (5)	0.0034 (4)	0.0025 (4)
C1	0.0240 (5)	0.0193 (5)	0.0199 (5)	−0.0003 (4)	0.0020 (4)	0.0011 (4)
C2	0.0258 (5)	0.0173 (5)	0.0215 (5)	−0.0005 (4)	0.0004 (4)	−0.0006 (4)
C3	0.0298 (6)	0.0222 (5)	0.0198 (5)	0.0002 (5)	0.0032 (5)	0.0012 (4)
C4	0.0324 (6)	0.0197 (5)	0.0274 (6)	−0.0006 (5)	0.0027 (5)	0.0033 (5)
C5	0.0341 (7)	0.0187 (5)	0.0289 (6)	0.0012 (5)	0.0029 (5)	−0.0017 (5)
C6	0.0300 (6)	0.0210 (5)	0.0228 (6)	0.0017 (5)	0.0037 (5)	−0.0021 (5)
C7	0.0302 (6)	0.0226 (6)	0.0209 (6)	−0.0012 (5)	0.0038 (5)	0.0029 (5)
C8	0.0375 (7)	0.0289 (7)	0.0227 (6)	−0.0030 (6)	0.0033 (5)	−0.0012 (5)
C9	0.0409 (8)	0.0219 (6)	0.0292 (7)	−0.0017 (6)	0.0016 (6)	−0.0021 (5)
C10	0.0349 (7)	0.0229 (6)	0.0268 (6)	−0.0034 (5)	0.0005 (5)	0.0030 (5)
C11	0.0247 (5)	0.0238 (5)	0.0218 (5)	−0.0027 (5)	−0.0009 (4)	0.0023 (5)
C12	0.0293 (6)	0.0307 (7)	0.0207 (6)	−0.0033 (5)	0.0006 (5)	0.0043 (5)
C13	0.0275 (6)	0.0346 (7)	0.0228 (6)	−0.0013 (5)	0.0025 (5)	−0.0005 (5)
C14	0.0346 (7)	0.0273 (6)	0.0313 (7)	0.0020 (6)	0.0062 (6)	−0.0014 (5)
C15	0.0356 (7)	0.0229 (6)	0.0291 (6)	0.0008 (5)	0.0051 (6)	0.0034 (5)
C16	0.0246 (5)	0.0234 (6)	0.0220 (6)	−0.0013 (4)	0.0012 (5)	0.0022 (5)
C17	0.0464 (9)	0.0483 (9)	0.0251 (7)	0.0006 (8)	0.0071 (6)	−0.0055 (7)

### 3-Chloro-2-nitrobenzoic acid–6-methylquinoline (1/1) (III) Geometric parameters (Å, º)

**Table:**

Cl1—C3	1.7316 (13)	C8—C9	1.401 (2)
O1—C7	1.3035 (17)	C8—H8	0.9500
O1—H1	0.847 (13)	C9—C10	1.372 (2)
O2—C7	1.2170 (18)	C9—H9	0.9500
O3—N1	1.2238 (18)	C10—C11	1.416 (2)
O4—N1	1.2189 (18)	C10—H10	0.9500
N1—C2	1.4797 (17)	C11—C16	1.4197 (18)
N2—C8	1.322 (2)	C11—C12	1.4217 (18)
N2—C16	1.3717 (17)	C12—C13	1.372 (2)
N2—H2	0.879 (14)	C12—H12	0.9500
C1—C6	1.3918 (18)	C13—C14	1.421 (2)
C1—C2	1.3986 (17)	C13—C17	1.507 (2)
C1—C7	1.5065 (18)	C14—C15	1.376 (2)
C2—C3	1.3890 (17)	C14—H14	0.9500
C3—C4	1.3899 (19)	C15—C16	1.4124 (19)
C4—C5	1.3900 (19)	C15—H15	0.9500
C4—H4	0.9500	C17—H17A	0.9800
C5—C6	1.3902 (19)	C17—H17B	0.9800
C5—H5	0.9500	C17—H17C	0.9800
C6—H6	0.9500		
			
C7—O1—H1	109 (3)	C10—C9—C8	118.97 (14)
O4—N1—O3	125.11 (13)	C10—C9—H9	120.5
O4—N1—C2	117.38 (12)	C8—C9—H9	120.5
O3—N1—C2	117.42 (13)	C9—C10—C11	119.86 (13)
C8—N2—C16	119.84 (12)	C9—C10—H10	120.1
C8—N2—H2	117 (4)	C11—C10—H10	120.1
C16—N2—H2	123 (4)	C10—C11—C16	117.81 (12)
C6—C1—C2	117.80 (11)	C10—C11—C12	123.23 (13)
C6—C1—C7	120.75 (11)	C16—C11—C12	118.95 (13)
C2—C1—C7	121.45 (11)	C13—C12—C11	121.31 (13)
C3—C2—C1	121.42 (12)	C13—C12—H12	119.3
C3—C2—N1	117.00 (11)	C11—C12—H12	119.3
C1—C2—N1	121.58 (11)	C12—C13—C14	118.75 (13)
C2—C3—C4	119.96 (12)	C12—C13—C17	121.67 (14)
C2—C3—Cl1	120.65 (10)	C14—C13—C17	119.56 (15)
C4—C3—Cl1	119.39 (10)	C15—C14—C13	121.68 (14)
C3—C4—C5	119.33 (12)	C15—C14—H14	119.2
C3—C4—H4	120.3	C13—C14—H14	119.2
C5—C4—H4	120.3	C14—C15—C16	119.71 (13)
C4—C5—C6	120.30 (12)	C14—C15—H15	120.1
C4—C5—H5	119.9	C16—C15—H15	120.1
C6—C5—H5	119.9	N2—C16—C15	119.72 (12)
C5—C6—C1	121.19 (12)	N2—C16—C11	120.72 (12)
C5—C6—H6	119.4	C15—C16—C11	119.55 (12)
C1—C6—H6	119.4	C13—C17—H17A	109.5
O2—C7—O1	125.02 (13)	C13—C17—H17B	109.5
O2—C7—C1	120.90 (12)	H17A—C17—H17B	109.5
O1—C7—C1	114.08 (12)	C13—C17—H17C	109.5
N2—C8—C9	122.77 (13)	H17A—C17—H17C	109.5
N2—C8—H8	118.6	H17B—C17—H17C	109.5
C9—C8—H8	118.6		
			
C6—C1—C2—C3	0.1 (2)	C2—C1—C7—O1	−176.81 (13)
C7—C1—C2—C3	−179.62 (13)	C16—N2—C8—C9	0.2 (2)
C6—C1—C2—N1	180.00 (13)	N2—C8—C9—C10	−1.2 (3)
C7—C1—C2—N1	0.25 (19)	C8—C9—C10—C11	0.8 (2)
O4—N1—C2—C3	83.25 (16)	C9—C10—C11—C16	0.6 (2)
O3—N1—C2—C3	−93.42 (16)	C9—C10—C11—C12	−178.41 (14)
O4—N1—C2—C1	−96.63 (16)	C10—C11—C12—C13	179.70 (14)
O3—N1—C2—C1	86.70 (17)	C16—C11—C12—C13	0.7 (2)
C1—C2—C3—C4	−0.4 (2)	C11—C12—C13—C14	1.2 (2)
N1—C2—C3—C4	179.77 (13)	C11—C12—C13—C17	−177.59 (14)
C1—C2—C3—Cl1	179.38 (10)	C12—C13—C14—C15	−1.8 (2)
N1—C2—C3—Cl1	−0.49 (18)	C17—C13—C14—C15	177.06 (15)
C2—C3—C4—C5	0.4 (2)	C13—C14—C15—C16	0.4 (2)
Cl1—C3—C4—C5	−179.37 (11)	C8—N2—C16—C15	−179.21 (15)
C3—C4—C5—C6	−0.2 (2)	C8—N2—C16—C11	1.3 (2)
C4—C5—C6—C1	0.0 (2)	C14—C15—C16—N2	−177.94 (14)
C2—C1—C6—C5	0.1 (2)	C14—C15—C16—C11	1.6 (2)
C7—C1—C6—C5	179.82 (13)	C10—C11—C16—N2	−1.7 (2)
C6—C1—C7—O2	−176.84 (16)	C12—C11—C16—N2	177.42 (13)
C2—C1—C7—O2	2.9 (2)	C10—C11—C16—C15	178.81 (14)
C6—C1—C7—O1	3.4 (2)	C12—C11—C16—C15	−2.1 (2)

### 3-Chloro-2-nitrobenzoic acid–6-methylquinoline (1/1) (III) Hydrogen-bond geometry (Å, º)

**Table:**

*D*—H···*A*	*D*—H	H···*A*	*D*···*A*	*D*—H···*A*
O1—H1···N2	0.85 (3)	1.72 (3)	2.5640 (17)	174 (3)
N2—H2···O1	0.88 (4)	1.69 (4)	2.5640 (17)	170 (4)
C5—H5···.O2^i^	0.95	2.44	3.3245 (19)	155
C8—H8···.O2	0.95	2.46	3.1438 (19)	129

Symmetry code: (i) *x*, *y*+1, *z*.

### 4-Chloro-2-nitrobenzoic acid–6-methylquinoline (1/1) (IV) Crystal data

**Table:**

C_7_H_3.48_ClNO_4_·C_10_H_9.52_N	*F*(000) = 712.00
*M**_r_* = 344.75	*D*_x_ = 1.433 Mg m^−^^3^
Monoclinic, *C**c*	Mo *K*α radiation, λ = 0.71075 Å
*a* = 7.4271 (6) Å	Cell parameters from 14736 reflections
*b* = 14.4348 (6) Å	θ = 3.1–30.2°
*c* = 16.2208 (7) Å	µ = 0.26 mm^−^^1^
β = 113.203 (3)°	*T* = 185 K
*V* = 1598.35 (16) Å^3^	Block, colorless
*Z* = 4	0.28 × 0.25 × 0.20 mm

### 4-Chloro-2-nitrobenzoic acid–6-methylquinoline (1/1) (IV) Data collection

**Table:**

Rigaku R-AXIS RAPIDII diffractometer	4158 reflections with *I* > 2σ(*I*)
Detector resolution: 10.000 pixels mm^-1^	*R*_int_ = 0.015
ω scans	θ_max_ = 30.0°, θ_min_ = 3.1°
Absorption correction: numerical (*NUMABS*; Higashi, 1999)	*h* = −10→10
*T*_min_ = 0.931, *T*_max_ = 0.949	*k* = −19→20
16695 measured reflections	*l* = −22→22
4645 independent reflections	

### 4-Chloro-2-nitrobenzoic acid–6-methylquinoline (1/1) (IV) Refinement

**Table:**

Refinement on *F*^2^	Secondary atom site location: difference Fourier map
Least-squares matrix: full	Hydrogen site location: mixed
*R*[*F*^2^ > 2σ(*F*^2^)] = 0.030	H atoms treated by a mixture of independent and constrained refinement
*wR*(*F*^2^) = 0.081	*w* = 1/[σ^2^(*F*_o_^2^) + (0.0529*P*)^2^ + 0.1089*P*] where *P* = (*F*_o_^2^ + 2*F*_c_^2^)/3
*S* = 1.09	(Δ/σ)_max_ < 0.001
4645 reflections	Δρ_max_ = 0.35 e Å^−^^3^
225 parameters	Δρ_min_ = −0.16 e Å^−^^3^
4 restraints	Absolute structure: Flack *x* determined using 1899 quotients [(*I*^+^)-(*I*^-^)]/[(*I*^+^)+(*I*^-^)] (Parsons *et al*., 2013)
Primary atom site location: structure-invariant direct methods	Absolute structure parameter: −0.023 (9)

### 4-Chloro-2-nitrobenzoic acid–6-methylquinoline (1/1) (IV) Special details

**Table:**

Geometry. All esds (except the esd in the dihedral angle between two l.s. planes) are estimated using the full covariance matrix. The cell esds are taken into account individually in the estimation of esds in distances, angles and torsion angles; correlations between esds in cell parameters are only used when they are defined by crystal symmetry. An approximate (isotropic) treatment of cell esds is used for estimating esds involving l.s. planes.

### 4-Chloro-2-nitrobenzoic acid–6-methylquinoline (1/1) (IV) Fractional atomic coordinates and isotropic or equivalent isotropic displacement parameters (Å^2^)

**Table:**

	*x*	*y*	*z*	*U*_iso_*/*U*_eq_	Occ. (<1)
Cl1	0.60068 (7)	0.04982 (4)	0.11442 (3)	0.04410 (14)	
O1	0.5629 (2)	0.26210 (10)	0.47141 (9)	0.0398 (3)	
H1	0.543 (10)	0.290 (4)	0.512 (3)	0.060*	0.48 (5)
O2	0.5224 (3)	0.38723 (10)	0.38416 (11)	0.0448 (4)	
O3	0.8822 (3)	0.13730 (13)	0.49214 (11)	0.0557 (5)	
O4	0.6225 (5)	0.05352 (14)	0.45694 (15)	0.0794 (8)	
N1	0.7212 (3)	0.11139 (11)	0.44056 (12)	0.0423 (4)	
N2	0.5616 (2)	0.36395 (11)	0.59693 (10)	0.0297 (3)	
H2	0.543 (8)	0.336 (3)	0.546 (2)	0.044*	0.52 (5)
C1	0.5733 (3)	0.23966 (12)	0.33064 (11)	0.0274 (3)	
C2	0.6451 (3)	0.14958 (13)	0.34899 (12)	0.0292 (3)	
C3	0.6568 (3)	0.09002 (13)	0.28407 (13)	0.0325 (4)	
H3	0.708220	0.029174	0.298910	0.039*	
C4	0.5909 (3)	0.12266 (15)	0.19705 (12)	0.0322 (4)	
C5	0.5203 (3)	0.21248 (15)	0.17566 (12)	0.0339 (4)	
H5	0.478690	0.234289	0.115740	0.041*	
C6	0.5111 (3)	0.26997 (13)	0.24225 (12)	0.0311 (4)	
H6	0.461428	0.331090	0.227361	0.037*	
C7	0.5518 (3)	0.30391 (14)	0.39964 (12)	0.0306 (4)	
C8	0.5951 (3)	0.45366 (13)	0.59630 (12)	0.0317 (4)	
H8	0.593525	0.481201	0.542828	0.038*	
C9	0.6328 (3)	0.50947 (13)	0.67162 (14)	0.0353 (4)	
H9	0.653792	0.574126	0.669007	0.042*	
C10	0.6390 (3)	0.46968 (13)	0.74905 (13)	0.0337 (4)	
H10	0.666629	0.506454	0.801199	0.040*	
C11	0.6041 (3)	0.37319 (12)	0.75145 (12)	0.0282 (3)	
C12	0.6091 (3)	0.32621 (16)	0.82941 (12)	0.0350 (4)	
H12	0.637854	0.360018	0.883369	0.042*	
C13	0.5730 (3)	0.23282 (16)	0.82796 (15)	0.0391 (4)	
C14	0.5312 (3)	0.18307 (15)	0.74760 (17)	0.0401 (4)	
H14	0.506395	0.118429	0.746653	0.048*	
C15	0.5253 (3)	0.22500 (13)	0.67141 (14)	0.0354 (4)	
H15	0.496495	0.189993	0.618127	0.042*	
C16	0.5625 (2)	0.32146 (13)	0.67232 (12)	0.0277 (3)	
C17	0.5805 (4)	0.1813 (2)	0.9099 (2)	0.0549 (6)	
H17A	0.456380	0.148651	0.896253	0.082*	
H17B	0.601873	0.225362	0.958894	0.082*	
H17C	0.688173	0.136438	0.928067	0.082*	

### 4-Chloro-2-nitrobenzoic acid–6-methylquinoline (1/1) (IV) Atomic displacement parameters (Å^2^)

**Table:**

	*U*^11^	*U*^22^	*U*^33^	*U*^12^	*U*^13^	*U*^23^
Cl1	0.0500 (3)	0.0567 (3)	0.0311 (2)	−0.0080 (2)	0.02182 (19)	−0.0170 (2)
O1	0.0611 (9)	0.0370 (7)	0.0260 (6)	0.0069 (7)	0.0223 (6)	−0.0011 (5)
O2	0.0707 (11)	0.0298 (7)	0.0430 (8)	−0.0030 (7)	0.0322 (8)	−0.0026 (6)
O3	0.0667 (11)	0.0593 (10)	0.0284 (7)	0.0180 (9)	0.0052 (7)	0.0040 (7)
O4	0.149 (2)	0.0547 (11)	0.0454 (10)	−0.0336 (13)	0.0493 (13)	0.0042 (9)
N1	0.0760 (13)	0.0273 (8)	0.0260 (7)	0.0026 (8)	0.0226 (8)	0.0006 (6)
N2	0.0320 (8)	0.0326 (7)	0.0246 (7)	0.0054 (6)	0.0113 (6)	−0.0020 (6)
C1	0.0325 (8)	0.0297 (8)	0.0230 (7)	−0.0069 (7)	0.0140 (6)	−0.0037 (6)
C2	0.0370 (9)	0.0307 (9)	0.0214 (7)	−0.0053 (7)	0.0133 (6)	−0.0006 (6)
C3	0.0390 (9)	0.0319 (9)	0.0300 (8)	−0.0065 (7)	0.0172 (7)	−0.0048 (7)
C4	0.0333 (9)	0.0428 (10)	0.0243 (8)	−0.0098 (7)	0.0154 (7)	−0.0110 (7)
C5	0.0347 (9)	0.0468 (10)	0.0216 (7)	−0.0056 (8)	0.0124 (7)	−0.0003 (7)
C6	0.0314 (9)	0.0365 (9)	0.0261 (8)	−0.0038 (7)	0.0122 (7)	0.0016 (7)
C7	0.0332 (9)	0.0344 (9)	0.0268 (8)	−0.0046 (7)	0.0145 (7)	−0.0047 (7)
C8	0.0341 (8)	0.0350 (9)	0.0271 (9)	0.0064 (7)	0.0133 (8)	0.0056 (7)
C9	0.0411 (10)	0.0260 (8)	0.0386 (9)	0.0021 (7)	0.0153 (8)	0.0004 (8)
C10	0.0389 (9)	0.0304 (8)	0.0304 (9)	0.0030 (7)	0.0120 (7)	−0.0064 (7)
C11	0.0293 (8)	0.0309 (8)	0.0249 (7)	0.0040 (6)	0.0113 (6)	−0.0001 (6)
C12	0.0363 (10)	0.0443 (10)	0.0258 (8)	0.0066 (8)	0.0137 (7)	0.0039 (8)
C13	0.0313 (9)	0.0474 (11)	0.0418 (10)	0.0093 (8)	0.0177 (8)	0.0178 (9)
C14	0.0342 (10)	0.0320 (9)	0.0546 (12)	0.0012 (8)	0.0180 (9)	0.0083 (9)
C15	0.0365 (10)	0.0286 (8)	0.0395 (10)	0.0007 (7)	0.0133 (8)	−0.0044 (7)
C16	0.0269 (8)	0.0289 (8)	0.0273 (8)	0.0037 (6)	0.0108 (6)	−0.0006 (7)
C17	0.0506 (13)	0.0653 (15)	0.0552 (14)	0.0110 (11)	0.0276 (11)	0.0320 (12)

### 4-Chloro-2-nitrobenzoic acid–6-methylquinoline (1/1) (IV) Geometric parameters (Å, º)

**Table:**

Cl1—C4	1.7273 (18)	C8—C9	1.397 (3)
O1—C7	1.285 (2)	C8—H8	0.9500
O1—H1	0.836 (15)	C9—C10	1.365 (3)
O2—C7	1.230 (3)	C9—H9	0.9500
O3—N1	1.217 (3)	C10—C11	1.420 (3)
O4—N1	1.208 (3)	C10—H10	0.9500
N1—C2	1.472 (2)	C11—C16	1.410 (2)
N2—C8	1.319 (2)	C11—C12	1.423 (3)
N2—C16	1.365 (2)	C12—C13	1.373 (3)
N2—H2	0.873 (14)	C12—H12	0.9500
C1—C6	1.392 (2)	C13—C14	1.411 (3)
C1—C2	1.392 (3)	C13—C17	1.505 (3)
C1—C7	1.510 (2)	C14—C15	1.361 (3)
C2—C3	1.389 (3)	C14—H14	0.9500
C3—C4	1.382 (3)	C15—C16	1.418 (3)
C3—H3	0.9500	C15—H15	0.9500
C4—C5	1.390 (3)	C17—H17A	0.9800
C5—C6	1.385 (3)	C17—H17B	0.9800
C5—H5	0.9500	C17—H17C	0.9800
C6—H6	0.9500		
			
C7—O1—H1	122 (5)	C10—C9—C8	118.99 (17)
O4—N1—O3	125.3 (2)	C10—C9—H9	120.5
O4—N1—C2	117.1 (2)	C8—C9—H9	120.5
O3—N1—C2	117.46 (18)	C9—C10—C11	119.82 (17)
C8—N2—C16	120.89 (15)	C9—C10—H10	120.1
C8—N2—H2	113 (4)	C11—C10—H10	120.1
C16—N2—H2	126 (4)	C16—C11—C10	118.13 (16)
C6—C1—C2	117.21 (16)	C16—C11—C12	118.45 (17)
C6—C1—C7	118.77 (16)	C10—C11—C12	123.42 (17)
C2—C1—C7	123.97 (16)	C13—C12—C11	121.08 (18)
C3—C2—C1	123.32 (17)	C13—C12—H12	119.5
C3—C2—N1	114.79 (16)	C11—C12—H12	119.5
C1—C2—N1	121.85 (16)	C12—C13—C14	119.07 (18)
C4—C3—C2	117.43 (18)	C12—C13—C17	122.1 (2)
C4—C3—H3	121.3	C14—C13—C17	118.9 (2)
C2—C3—H3	121.3	C15—C14—C13	121.93 (19)
C3—C4—C5	121.32 (17)	C15—C14—H14	119.0
C3—C4—Cl1	118.61 (16)	C13—C14—H14	119.0
C5—C4—Cl1	120.06 (14)	C14—C15—C16	119.38 (19)
C6—C5—C4	119.59 (16)	C14—C15—H15	120.3
C6—C5—H5	120.2	C16—C15—H15	120.3
C4—C5—H5	120.2	N2—C16—C11	119.95 (16)
C5—C6—C1	121.10 (18)	N2—C16—C15	119.96 (16)
C5—C6—H6	119.4	C11—C16—C15	120.08 (17)
C1—C6—H6	119.4	C13—C17—H17A	109.5
O2—C7—O1	125.94 (17)	C13—C17—H17B	109.5
O2—C7—C1	120.75 (16)	H17A—C17—H17B	109.5
O1—C7—C1	113.29 (16)	C13—C17—H17C	109.5
N2—C8—C9	122.19 (16)	H17A—C17—H17C	109.5
N2—C8—H8	118.9	H17B—C17—H17C	109.5
C9—C8—H8	118.9		
			
C6—C1—C2—C3	0.0 (3)	C16—N2—C8—C9	0.0 (3)
C7—C1—C2—C3	177.39 (18)	N2—C8—C9—C10	1.3 (3)
C6—C1—C2—N1	177.83 (18)	C8—C9—C10—C11	−1.0 (3)
C7—C1—C2—N1	−4.8 (3)	C9—C10—C11—C16	−0.4 (3)
O4—N1—C2—C3	−73.6 (3)	C9—C10—C11—C12	179.57 (18)
O3—N1—C2—C3	102.9 (2)	C16—C11—C12—C13	−0.5 (3)
O4—N1—C2—C1	108.3 (2)	C10—C11—C12—C13	179.59 (19)
O3—N1—C2—C1	−75.1 (3)	C11—C12—C13—C14	0.3 (3)
C1—C2—C3—C4	−0.9 (3)	C11—C12—C13—C17	179.03 (19)
N1—C2—C3—C4	−178.83 (17)	C12—C13—C14—C15	−0.1 (3)
C2—C3—C4—C5	1.6 (3)	C17—C13—C14—C15	−178.90 (19)
C2—C3—C4—Cl1	−179.20 (14)	C13—C14—C15—C16	0.1 (3)
C3—C4—C5—C6	−1.6 (3)	C8—N2—C16—C11	−1.4 (3)
Cl1—C4—C5—C6	179.26 (14)	C8—N2—C16—C15	179.73 (18)
C4—C5—C6—C1	0.7 (3)	C10—C11—C16—N2	1.6 (3)
C2—C1—C6—C5	0.1 (3)	C12—C11—C16—N2	−178.32 (16)
C7—C1—C6—C5	−177.44 (17)	C10—C11—C16—C15	−179.54 (18)
C6—C1—C7—O2	−16.2 (3)	C12—C11—C16—C15	0.5 (3)
C2—C1—C7—O2	166.48 (19)	C14—C15—C16—N2	178.48 (17)
C6—C1—C7—O1	162.34 (17)	C14—C15—C16—C11	−0.4 (3)
C2—C1—C7—O1	−15.0 (3)		

### 4-Chloro-2-nitrobenzoic acid–6-methylquinoline (1/1) (IV) Hydrogen-bond geometry (Å, º)

**Table:**

*D*—H···*A*	*D*—H	H···*A*	*D*···*A*	*D*—H···*A*
O1—H1···N2	0.84 (7)	1.70 (6)	2.514 (2)	163 (7)
N2—H2···O1	0.87 (4)	1.67 (5)	2.514 (2)	162 (4)
C10—H10···O2^i^	0.95	2.54	3.364 (3)	145

Symmetry code: (i) *x*, −*y*+1, *z*+1/2.
